# Design and Additive Manufacturing of Porous Sound Absorbers—A Machine-Learning Approach

**DOI:** 10.3390/ma14071747

**Published:** 2021-04-01

**Authors:** Sebastian Kuschmitz, Tobias P. Ring, Hagen Watschke, Sabine C. Langer, Thomas Vietor

**Affiliations:** 1 TU Braunschweig, Institute for Engineering Design, 38106 Braunschweig, Germany; h.watschke@tu-braunschweig.de (H.W.); t.vietor@tu-braunschweig.de (T.V.); 2 TU Braunschweig, Institute for Acoustics, 38106 Braunschweig, Germany; s.langer@tu-braunschweig.de

**Keywords:** acoustics, porous materials, material extrusion, design for additive manufacturing, sound absorption, artificial neural network, machine learning

## Abstract

Additive manufacturing (AM), widely known as 3D-printing, builds parts by adding material in a layer-by-layer process. This tool-less procedure enables the manufacturing of porous sound absorbers with defined geometric features, however, the connection of the acoustic behavior and the material’s micro-scale structure is only known for special cases. To bridge this gap, the work presented here employs machine-learning techniques that compute acoustic material parameters (Biot parameters) from the material’s micro-scale geometry. For this purpose, a set of test specimens is used that have been developed in earlier studies. The test specimens resemble generic absorbers by a regular lattice structure based on a bar design and allow a variety of parameter variations, such as bar width, or bar height. A set of 50 test specimens is manufactured by material extrusion (MEX) with a nozzle diameter of 0.2 mm and a targeted under extrusion to represent finer structures. For the training of the machine learning models, the Biot parameters are inversely identified from the manufactured specimen. Therefore, laboratory measurements of the flow resistivity and absorption coefficient are used. The resulting data is used for training two different machine learning models, an artificial neural network and a k-nearest neighbor approach. It can be shown that both models are able to predict the Biot parameters from the specimen’s micro-scale with reasonable accuracy. Moreover, the detour via the Biot parameters allows the application of the process for application cases that lie beyond the scope of the initial database, for example, the material behavior for other sound fields or frequency ranges can be predicted. This makes the process particularly useful for material design and takes a step forward in the direction of tailoring materials specific to their application.

## 1. Introduction

Porous materials are widely used for sound absorption and noise mitigation and find applications in various technical disciplines. Therein, three mechanisms can be distinguished: absorption of airborne sound, decoupling of vibrating systems and reduction of flow-induced sound. First, the most common and well-known application for porous materials is sound absorption, for example in room acoustics [1] or porous liners in aircraft engine inlet and exhaust pipe [2,3]. The scope of this work as well lies within this field. For this mechanism, the dominating effect is the dissipation of the acoustic energy when the material is placed in front of an acoustically rigid boundary. A second important aspect is the application of porous materials with the effect of decoupling vibrating structures. This effect is commonly employed for improving sound insulation of double panel walls, for example in aircraft fuselage sidewall panels [4,5] or in building acoustics [6,7]. Thereby, the two wall panels are separated by the porous material. The damped propagation of the acoustic wave inside the porous material results in a decrease of the mechanic coupling of the two panels. The effect of decoupling can be exploited as well with regard to aircraft structures. Here, the application of porous materials on the outer aircraft skin can reduce the excitation of structure-borne sound that results from an external pressure field [8]. The third application of porous materials is the reduction of flow-induced sound emitted from moving objects [9,10,11,12]. All mentioned applications make use of sheets of porous materials, for example, fibrous mineral wool, synthetic foam, or porous metals. These porous materials show favorable acoustic properties, specifically a high absorption coefficient, especially in the higher frequency regime [13]. Nevertheless, these materials typically consist of an irregular microstructure and a geometric description of the microstructure is available as homogenized values only. This makes an investigation of the material structure on the micro-scale (the micro-scale is hereafter understood as the size scale of the pores, see [14], whereas the macro-scale are the overall specimens dimensions) and its relation to the acoustic behavior rather difficult. Therefore, the basic idea of the presented paper is to bridge the gap between the absorber’s micro-scale geometry and its acoustic behavior by means of machine-learning techniques. The acoustic behavior thereby is described material-specific parameters in combination with mechanical porous media models. This “detour” allows the necessary input data set to be rather small and enables the prediction of the acoustic behavior for arbitrary application cases of the material that go beyond the specific test cases with which the data has been retrieved.

### 1.1. Description of Porous Media by Mechanical Models

A common engineering task in acoustics is the adjustment of the appropriate amount of damping of a fluid cavity, for example to adjust reverberation time. Acoustic damping can be effectively reached by means of porous materials that are mounted onto the surface of a rigid wall and thus serve as acoustic absorbers. The general procedure for the application of porous material commonly is a first laboratory-scale characterization of the material followed by an analysis of the target system, resulting in information where and how the material at hand can effectively be employed. The laboratory tests mostly incorporate acoustic measurements of the absorption coefficient, flow resistivity and further parameters. This straightforward procedure allows for a reasonable application of available materials. Nevertheless, the possibility to directly design materials, in the sense of tailoring them with special regard to the application often is of interest. An approach to determine the necessary acoustic parameters that fulfill a given task is shown in [15] by tailoring porous media to mitigate aeroacoustic trailing edge noise. Nevertheless, a core challenge during the material design task of porous media is the difference between the geometric and acoustics properties of the material. On one side, the geometric description is essential for a subsequent manufacturing process. For porous media in acoustic applications, the geometry on both the macro-scale and the micro-scale is important. Geometric properties are, for example, the sample thickness on the macro-scale and the pore diameter (if the pores are assumed to be rather cylindrical) or even more complex descriptions of the pore geometry on the micro-scale. On the other side, the desired material properties are derived using mechanical models that resemble the acoustic behavior of the material. Thereby, the Biot model, [16,17], is the most complex one since it models the wave propagation of elastic waves within the material of both, the pressure wave in the fluid phase and the shear and pressure wave in the elastic (skeleton) phase of the material. Due to this full description, the Biot model is accepted as a reference for modeling porous materials [18]. Other, more simple models neglect the elasticity of the skeleton phase and model only the pressure wave within the fluid. They incorporate the influence of the skeleton by definition of equivalent fluid properties. These models are referred to as the class of equivalent fluid models. Prominent examples are the model introduced by Johnson et al. [19] and the model by Champoux and Allard [20]. All aforementioned models employ material parameters that represent the material in a homogenized way, the pore structure of the material is not resolved. These acoustic material parameters are hereafter referred to as Biot parameters. The Biot parameters can be classified into the parameters that can be attributed to the (equivalent) fluid phase and those attributed to the skeleton. The relevant parameters of the fluid phase are the porosity ϕ, the flow resistivity Ξ, the tortuosity $\alpha_{\infty }$ and the viscous and thermal characteristic lengths Λ, $\Lambda^{\prime }$. For the special case of the Johnson–Champoux–Allard–Lafarge (JCAL) model [19,20,21] that is used within this work, the static thermal permeability $k_{0}^{\prime }$ is additionally introduced. For the full description of the material using Biot’s theory, the mechanical parameters of the skeleton material are required as well. From the set of Biot parameters the flow resistivity, porosity and tortuosity can be easily explained: the porosity refers to the share of fluid volume on the overall sample volume, the flow resistivity measures the possibility of a fluid flow to pass through the material and the tortuosity indicates the degree of the geometric complexity of the pore structure. However, one of the major drawbacks of the porous material models is that at least some of the required parameters are hard to determine [18]. Indeed, some parameters can be measured, such as flow resistivity, porosity and tortuosity. Moreover, for a given material it is possible to inversely estimate these parameters, as shown for example in [22,23]. For the case of an available geometric description of the material on the micro-scale, in [24] the computation of the flow resistivity of a porous sample is computed using the Lattice–Boltzmann method. Nevertheless, in general, no connection between the Biot parameters and the micro-scale geometry is known. Therefore the application of porous material models is limited to materials that already exist and for which the required parameters can be measured or otherwise determined.

### 1.2. Additive Manufacturing of Porous Materials for Sound Absorption

One promising possibility to generate acoustically effective materials is the rapidly growing field of additive manufacturing (AM), also known as 3D printing. The layer-by-layer working principle of additive manufacturing processes provides new kinds of design freedom during product development. In this way, for example, undercuts, mesoscopic lattice structures, or free-form surfaces can be realized, making additive manufacturing particularly suitable for the production of acoustically effective structures [25]. The specimens investigated in this contribution consist of a micro-lattice of parallel bars as described in [26]. Hence, AM is employed within this contribution for manufacturing the required amount of specimens.

Due to the aforementioned reasons, AM provides the opportunity to significantly improve both geometric and functional product properties [27,28]. Due to the aforementioned potentials, AM processes can greatly improve the production of acoustically effective structures. Especially the material extrusion (MEX) is predestined for this application due to its process principles, for example, no need for support structures, no requirement for removing powder or resin in undercuts and channels, or the use of fine nozzle diameters. The high resolution of the microstructure in the x-y direction is only limited by the nozzle diameter, which in MEX processes can be down to 0.1 mm on the lower end, and the printer itself. Other processes such as the powder bed fusion of polymers (PBF-P) or vat photopolymerization (VAT) theoretically also offer the possibility of producing even finer microstructures. However, since the liquid material of the VAT process (capillary effects, etc.) leads to unwanted hardening of the material and the powder bed in the PBF-P process results in sintering as well as powder residues in the geometry, the MEX process is particularly well suited for this use [27].

In the past, AM techniques have been used to produce a variety of sound-absorbing materials, including Helmholtz resonators [29], straight [30], inclined [31], angled tubes [29], sound crystals [32]. Moreover, microperforated plates [33] and sounds absorbers made of micro-grids [34,35,36] have been manufactured (see also [26,34,37,38,39,40]). The additive manufacturing of porous absorbers has also been addressed in a rudimentary way in the literature. Ring et al. have demonstrated the additive manufacturing of the porous and acoustically effective absorber structures using MEX processes and were able to show the potential of these structures. Additionally, the authors were able to investigate relationships between the geometry and acoustic properties, the Biot parameters (tortuosity, porosity, etc.) [26,41]. Zielinski et al. have performed a 3D printing process comparison with different materials using porous absorbers, revealing that the process parameters have a crucial influence on the reproducibility of acoustically effective structures [37]. Boulvert et al. have presented studies on geometrical factors influencing the acoustic properties, in which mainly the gradation of porosity is realized via the infill of the porous absorbers [38]. This approach is similar to the one shown in [26], where grading the porosity via the infill alone does not fully exploit the design potential of additive manufacturing.

A common feature of all previously presented research results is the focus on the technical feasibility of additive manufacturing of acoustically effective porous structures and research into factors influencing the acoustic properties. However, the focus so far has been only on the manufacturing of porous geometry without defining the acoustic properties in advance. Nevertheless, the use of AM technologies for the creation of porous structures offers the possibility to efficiently introduce acoustic properties in a product-specific way, taking into account the potentials and limitations of the manufacturing process. A method for targeted adjustment of acoustic properties through defined geometries, taking into account process-related influencing factors, thus is desirable.

### 1.3. Aims and Scope of the Presented Work

As mentioned above, several experimental capabilities exist to investigate the acoustic behavior of porous materials. Moreover, the acoustic behavior of porous materials can be described using various mechanical models of different complexity and accuracy. Recently, AM capabilities have proven successful in manufacturing generic porous materials as well. Nevertheless, for manufacturing a porous specimen, a suitable geometric description of the specimen is required on both, the micro- and macro-scale. The acoustic description of the specimen with both, experiments and/or mechanical models, involves the description with the Biot parameters. These two “worlds” (macro-/micro-scale of the geometry and Biot parameters) are rather detached, as no general connection of geometric dimensions and the Biot parameters of porous materials is known. Indeed, for some special cases models exist to estimate the Biot parameters from the specimen geometry, for example, the tortuosity can be estimated from the porosity as shown in [14,42,43,44]. In order to bridge this gap, the work presented here employs Machine-Learning (ML) techniques to build a model that computes the Biot parameters for a given geometric description of a specimen.

A similar idea, the computation of the acoustic behavior of porous material using ML techniques, is presented, for example, in [45,46,47]. In contrast to the approach proposed within this contribution, the authors directly compute the absorption coefficient of the porous material using a neural network model. This approach is reasonable as long as the macroscopic sample dimensions are kept constant or if data from samples with different dimensions are available for the training, whereas the latter is the case in the mentioned publications. Another approach is presented in [48]. Here the authors use more material-specific parameters for prediction of the absorption coefficient of a porous absorber. However, the parameter set chosen is rather general and accounts for the type of material only by referring to the porosity. For the work presented here, a “detour” via the Biot parameters is used. It is a detour in the sense that the whole process becomes more complex, as the Biot parameters have to be derived which is a challenging process involving ill-posed mathematical problems. However, this detour has some favorable implications: the Biot parameters are material-specific parameters. This way, no mixing of the description of the material and its acoustic behavior (for example: absorption coefficient in front of a rigid wall) occurs. Having the Biot parameters available, it becomes possible to compute the acoustic behavior of the investigated material for conditions apparent in the available data (sound field, macro-scale dimensions of the specimen), but other conditions can be covered as well. For example, the behavior of the material for other frequency ranges than the measured ones can be computed. Another application example for the Biot parameters is the case of more complex simulations, such as finite element computations. Using such wave-resolving methods, the behavior of the material can be evaluated in other sound fields, such as diffuse fields. Moreover, the dimension of the necessary training data is reduced as samples with a constant thickness can be used rather than a population comprising different sample thicknesses. Due to these reasons, the “detour” via the Biot parameters is assumed to justify the higher effort and adds value to this field of research.

The resulting ML models are then used for an inverse material design approach. Therefore, materials are designed that are supposed to show a predefined acoustic behavior. Thereby, the advantage of using the Biot parameters as a basis becomes clear, as this procedure can be done by adjusting the specimen geometry on the micro-scale only but as well on the macro-scale.

The procedure presented within this work allows to adjust the acoustic properties of the absorbers before production and thus enables an efficient application of acoustically effective materials that are best suited for the requirements of the application. In turn, this procedure might even reduce secondary factors such as costs, weight, or carbon footprint.

### 1.4. Outline of the Paper

The paper is organized as follows: in Section 2 the methods applied within this work are presented and a description of the developed specimen is given. A general overview of the applied procedure is presented in Section 2.1, the design methodology and manufacturing process for the generic specimens are shown in Section 2.2 and Section 2.3, respectively. The methods to acoustically investigate and derive Biot parameters are described in Section 2.4 and Section 2.5, the applied machine learning process including the applied models is described in Section 2.6. In Section 3, the results of the acoustical inspection, parameter identification and machine learning process are shown in Section 2.4 and Section 3.2. The results of the application of the process to generate new absorbers with a predefined behavior are shown in Section 3.3 and Section 3.4. A conclusion and an outlook is given in Section 4.

## 2. Materials and Methods

In this section, the study design and used methods are presented. The goal of the work is to build a model using ML techniques that computes the Biot parameters for a given specimen micro-scale geometry. In a subsequent step, the obtained ML models are used to design new sound-absorbing structures that exhibit a predefined acoustic behavior. Within this section, first, the overall concept of the process is described, followed by a description of the generic specimen investigated here. Furthermore, the used experimental setups for manufacturing and characterization of the specimens are described and the process to identify the Biot parameters is shown. Finally, the used machine learning models and the material design approach are described.

### 2.1. General Description of the Applied Process

**Figure 1:**
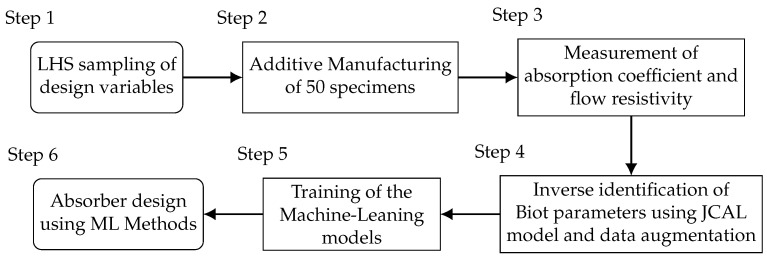
General procedure of the work to obtain ML models that predict Biot parameters from specimen geometry and their application to absorber design.

The goal to build ML models that allow the computation of Biot parameters from a geometric description of the specimens and to utilize these for material design employs a 6-step procedure. The process is sketched in Figure 1.

The generic specimens investigated within this work are presented in Section 2.2 and are parametrically defined by four so-called design variables. The values of the design variables are referred to as the design parameters and form the geometric description of the specimen on the micro-scale.

The procedure starts in Step 1 (see Section 2.2) with a Latin Hypercube Sampling (LHS) of the design variables to generate a population of 50 specimens that explore the chosen data range of the design variables. All 50 designs are shown as well in Table A1 in the Appendix A. In Step 2, the 50 designed specimens are manufactured using AM technology, this is described in detail in Section 2.3. With the design parameters, the required inputs for the ML models are available. Hence, the Biot parameters that form the outputs of the ML models have to be determined. It would be desirable to directly measure these quantities. However, only a few of the Biot parameters can be measured directly without tremendous effort. To the author’s knowledge, these are the flow resistivity, the porosity and the tortuosity. Since the authors only have access to a measurement setup for the flow resistivity, an inverse parameter identification procedure is employed for the remaining Biot parameters. Therefore, in Step 3 the flow resistivity and the absorption coefficient are measured, the measurement principles are described in Section 2.4. Using these quantities, the remaining Biot parameters are inversely identified in Step 4. Therefore, the specimen is modeled using the Johnson–Champoux–Allard–Lafarge (JCAL) model, this procedure is described in detail in Section 2.5. Within this step, a data augmentation procedure is employed as well in order to enrich the database. Now both, the micro-scale geometry (the design parameters) and the Biot parameters are available. These data sets form the training data for the ML models (inputs/features: design parameters, outputs/labels: Biot parameters) that are set up and trained in Step 5. This is described in Section 2.6. Thereby, two different models, a K-Nearest Neighbor model and an Artificial Neural network, are employed. Having these models available, the design of porous materials (Step 6) becomes possible. Therefore, an inverse procedure is employed that is described in Section 3.3.

### 2.2. Specimen Design (Step 1)

For the additive manufacturing of porous absorber structures, a test specimen was developed within the scope of the research work, taking design for additive manufacturing regarding design potentials and limitations into account. Detailed descriptions of the specimens baseline design can be found in [26]. For the development of AM parts, a systematic development with the aid of different design tools is important to encourage the generation of ideas and to ensure a goal-oriented design [28,49,50]. However, the application of AM potentials must be considered in the early stages of product development, otherwise, the freedoms of AM cannot be fully utilized [51]. For this reason, the limitations and potentials of additive manufacturing with a focus on the porosity of the test specimens were considered during development to ensure additive manufacturability. In addition to the manufacturability of the test specimens by material extrusion, the focus is also on the possibility to vary the test specimens to be able to manufacture different variants that show only a small variation of the absorption properties. For this reason, a regular lattice structure based on a bar design was selected that functions as a porous structure.

**Figure 2:**
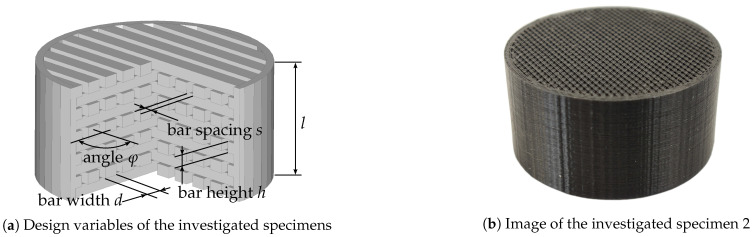
Overview of investigated specimen. (**a**) shows the test specimen including the design variables (from [26]) which are used to vary the test specimen. (**b**) shows the additive manufactured specimen 2.

This test specimen, as shown in Figure 2, is designed in layers and contains a defined number of bars per layer. The specimen design can generally be adjusted on the micro- and macro-scale. The micro-scale can be varied by different parameters, hereafter referred to as design variables. These are the bar width (*d*), the bar spacing (*s*), the bar height (*h*) and the plane angle (φ) and can be adjusted in a certain interval, see Table 1. Therein, the values are already chosen accordingly to the chosen AM process, here MEX. Table 1 shows that, for example, the bar spacing or the bar width can be varied in an interval of 0.10–0.50 mm, resulting in an increase or decrease of the bar number in the test specimen. All test specimens are printed with four outline shells resulting in a boundary of 0.80 mm with an extrusion width of 0.20 mm. The specimen diameter of 30 mm and height l=15 mm describe the specimen on the macro-scale. These dimensions are kept constant throughout this work. The specimen height is chosen in order to balance out the required production time (proportional to the height) and the ability to generate a reasonable amount of absorption in the frequency range that is chosen for this work. The frequency range and specimen diameter are prescribed by the available impedance tube with 900–6600 Hz (see Section 2.4 for more details).

In the scope of this contribution, a large number of different test specimens are manufactured. They are expected to exhibit a broad range of varying acoustic properties to link or identify the relationship of the geometry parameters with the acoustic parameters using a machine learning approach. This way, acoustic parameters should be able to be determined during the design phase.

The parameter selection of the design variables is carried out within the parameter limits mentioned in Table 1 and employs a Design of Experiment (DOE) methodology to ensure the meaningful selection of the design parameters. The DOE-process employs a Latin Hypercube Sampling (LHS) [52,53,54,55,56,57,58] strategy for this purpose. Within the scope of the contribution, the sample size amounts to 50 porous absorber structures, whose parameter combinations can be found in Table A1 in the Appendix A. The data generation was done by using the software OpenSCAD, see also [59].

### 2.3. Additive Manufacturing of the Specimens (Step 2)

The acoustically effective porous structures were manufactured through material extrusion (MEX). Material extrusion processes offer good possibilities for the production of porous absorber structures since extremely filigree structures can be produced through changeable nozzle diameters. It is assumed that this procedure offers the highest potential that the best possible acoustic properties can be realized. However, the production of porous absorber structures also places special demands on the manufacturing process, since the use of fine nozzle diameters (down to 0.10 mm) in combination with the realization of thin walls, gaps and overhangs is at the limit of what is practically possible. For this reason, the selection and controllability of the process parameters are essential for the successful production of acoustically effective structures. If the ambient conditions (ambient temperature, humidity) or the process parameters (flow, cooling, retraction distance) are incorrect, manufacturing errors will occur, leading to dimensional deviation of the part or termination of production due to clogging of the nozzle. The material used is polylactic acid (PLA) from DasFilament (Emskirchen, Germany) to create porous absorbers [26,41]. PLA is particularly suitable for the production of microstructures by material extrusion because it has comparatively low shrinkage and thus low thermally induced stresses. In addition, PLA’s lower printing temperature allows a fast cooling down of the extruded strands and makes it more suitable for parts with fine details such as overhangs and gaps, respectively, which is required for the bar design [60].

In addition, compared to other processes, such as PBF-P or VAT, there is the advantage that there is no material buildup due to sintering or unwanted polymerization due to capillary effects [27]. A total of 50 different test specimens were created as described in Section 2.2 which is similar to the amount of specimen in [47]. The resulting building times are approx. 120–240 min per specimen, depending on the number of bars and the layer height. The specimens were sliced using Simplify3D^®^ (4.1.2, Simplify3D, LLC, Cincinnati, OH, USA, 2020) and manufactured on the X400, a pro-consumer additive manufacturing machine from German RepRap GmbH (Feldkirchen, Germany), whose original extruders were replaced by E3D Hemera (Direct Extruder, 24 V). A 0.20 mm Micro-Swiss nozzle (Ramsey, MN, USA) was used as the extrusion nozzle. The process parameters were used as listed in Table 2.

**Figure 3:**
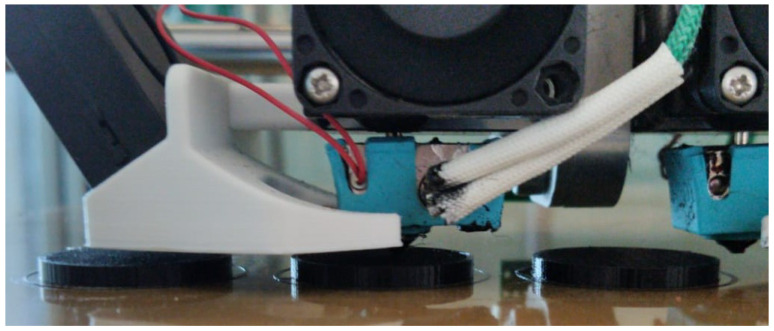
Additive manufacturing of three test specimens using material extrusion. Used 3D printer: X400, ppro-consumer additive manufacturing machine from German RepRap GmbH.

Figure 3 shows the printing process of three specimens. The first grid layer was applied horizontally to the printer because this orientation showed less detachment from the build platform. The smaller bar widths (<0.20 mm) were achieved by targeted under extrusion because this ensured a more continuous printing process. With smaller nozzle diameters, the nozzle got clogged or the flow was not constant. Since all samples were to be produced with as far as possible the same process parameters, the 0.20 mm nozzle was chosen. The environmental conditions (temperature and humidity) were constant during the manufacturing of the test specimens and in addition, the material is dried at 45 ${}^{\circ }C$ for about 6 h before processing. To assess the manufacturing accuracy of the additively manufactured test specimens, an actual-target comparison of the initial geometry and the manufactured test specimen was carried out (see Section 3.1.1).

### 2.4. Acoustical Investigation of Specimen Population (Step 3)

The method presented within this work aims to train ML models to map between the geometry and the Biot parameters of porous samples. The geometry of all manufactured specimens is known from the manufacturing process. Indeed, this manufacturing process is somewhat uncertain, hence the manufactured geometry probably differs from the design parameters. Nevertheless, previous studies have shown that the manufacturing process applied here at least allows a sufficient precision for the purpose of this work [26]. Apart from the specimen geometry, the Biot parameters are of interest and have to be determined. However, only few of the Biot parameters can be measured directly and only the measurement of the flow resistivity is available to the authors, the remaining Biot parameters are inversely identified, a procedure already successfully applied in the past. Therefore, the absorption coefficient and the flow resistivity of all specimens are measured and serve as inputs to the inverse procedure. In the following sections, the measurements of the flow resistivity (Section 2.4.1) and absorption coefficient (Section 2.4.2) are described. The inverse procedure to estimate the Biot parameters from the measurements in described in Section 2.5.

#### 2.4.1. Measurement of the Flow Resistivity

**Figure A1:**
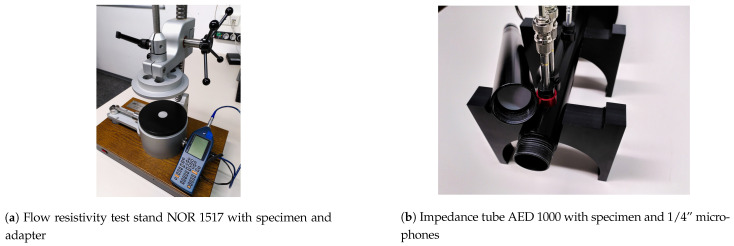
Measurement test stands used within this work.

All 50 manufactured specimens, as described in Section 2.2, are measured in the laboratory regarding their flow resistivity and their absorption coefficient. For the measurement of the flow resistivity, the method with the alternating flow is employed according to ISO 9053-2:2020 [61]. The measurement setup Nor1517A of Norsonic (Oelde-Stromberg, Germany) is used (see Figure A1a). It comprises a vessel that is equipped with a sinusoidally moving piston for generating an alternating pressure in the vessel and a measurement microphone to measure the pressure. The vessel is closed by the specimen, hence a pressure drop over the specimen can be measured. As the movement of the piston generates a known air flow *q* and the pressure drop over the specimen Δp is measured by the microphone, the airflow resistance *R* can be directly evaluated by $R=\frac{\Delta p}{q}$. The flow resistivity Ξ is computed from the airflow resistance with the specimen’s cross-sectional area *A* and the specimen thickness *l* by $\Xi =\frac{R\;A}{l}$. It should be noted that the specimen diameter used here is only 30 mm, whereas the measurement standard requires a diameter of 100 mm. To circumvent this difference, an adapter is manufactured and the measured values are corrected accordingly.

#### 2.4.2. Measurement of the Absorption Coefficient

The absorption coefficient is measured in an impedance tube with a diameter of 30 mm using the two-microphone method according to ISO 10534-2:1998 [62]. The measurement device is an AED 1000 AcoustiTube (Dresden, Germany) (see Figure A1b). Thereby, the acoustic transfer function between the two microphones is evaluated while the system is excited by a broadband white noise signal. From the transfer function, the complex reflection factor of the specimen surface can be evaluated and the absorption coefficient can be computed from the reflection factor. Due to the measurement principle, below the so-called cut-on frequency (here: 6600 Hz) that itself relates to the tube’s diameter, only plane waves travel through the tube. Thus, the presented absorption coefficients within this work are valid for normal incident plane waves only. As the cut-on frequency gives the upper limit for the possible frequency range, the lower limit is determined by the microphone distance (here: 20 mm) and results for the setting used here to 900–6600 Hz. Measurements in the frequency range below 900 Hz are assumed here to be unnecessary, as the absorption coefficient of a specimen with a height of l=15 mm is expected to be negligibly low.

### 2.5. Inverse Parameter Identification Using the JCAL-Model (Step 4)

Based on the measured flow resistivity and absorption coefficient data described in Section 2.4, the remaining Biot parameters of the porous specimens are inversely identified. This inverse procedure is common in the literature and has been applied often in the past, for example [23,26]. Therefore, the absorbers are modeled using the JCAL model [19,20,21]. The mathematical form of the model is used as given in [63] and shown in the appendix, see Appendix D. The JCAL model belongs to the class of equivalent fluid models, as the skeleton phase of the two-phase (fluid-filled pores and solid skeleton) system is assumed to be rigid. In former studies, the Biot model had been employed directly within this parameter identification schema [26], using an in-house finite-element code capable of using Biot’s model for numerical computations [64]. Nevertheless, numerical experiments have shown that the skeleton’s elasticity of the specimens used here can be neglected and the skeleton can be modeled as rigid. This allows the application of models belonging to the class of equivalent fluids, here the JCAL model. The JCAL model is a six parameter model, featuring the following parameters: the flow resistivity Ξ, the porosity ϕ, the high-frequency limit of the tortuosity $\alpha_{\infty }$, the viscous and thermal characteristic lengths Λ, $\Lambda^{\prime }$ and the static thermal permeability $k_{0}^{\prime }$.

The JCAL model allows the computation of the equivalent density $\tilde{\rho }$ and the equivalent bulk modulus $\tilde{K}$ of the equivalent fluid that represents the porous material, see Equations (A1) and (A2), respectively. Using these quantities, the characteristic impedance ${\tilde{Z}}_{c}$ and the complex wavenumber $\tilde{k}$ can be computed using the Equations (1) and (2) from [23], respectively.

(1) $$\begin{array}{cc} {\tilde{Z}}_{c} & =\sqrt{\tilde{\rho }\;\tilde{K}} \end{array}$$

(2) $$\begin{array}{cc} \tilde{k} & =\omega \sqrt{\frac{\tilde{\rho }}{\tilde{K}}} \end{array}$$

With the quantities, ${\tilde{Z}}_{c}$ and $\tilde{k}$, the surface impedance $Z_{s}$ of the porous material in front of a rigid impervious wall can be computed for the case of normal incident plane waves using Equation (3) from [65]. Thereby, the thickness of the specimen *l* is used, $j=\sqrt{-1}$.

(3) $$Z_{s}=-j\frac{{\tilde{Z}}_{c}}{\phi \;tan\left(\tilde{k}\;l\right)}$$

Here, it becomes clear why the prediction of the Biot parameters is preferred over the direct prediction of the absorption coefficient. The Biot parameters (or the subset used here for the JCAL model) are material-specific parameters. The acoustic behavior of the material for the case of a material mounted in front of a rigid wall is accounted for in subsequent computation. Hence, the process proposed here finally allows the description of the material at hand, independent of the mounting conditions or the sample thickness. Using the surface impedance $Z_{s}$ and the characteristic impedance of the surrounding fluid $Z_{0}$, the absorption coefficient α of the porous material can be computed:

(4) α=4ReZsZ0(Re(Zs)+Z0)2+Im(Zs)2

**Figure 4:**
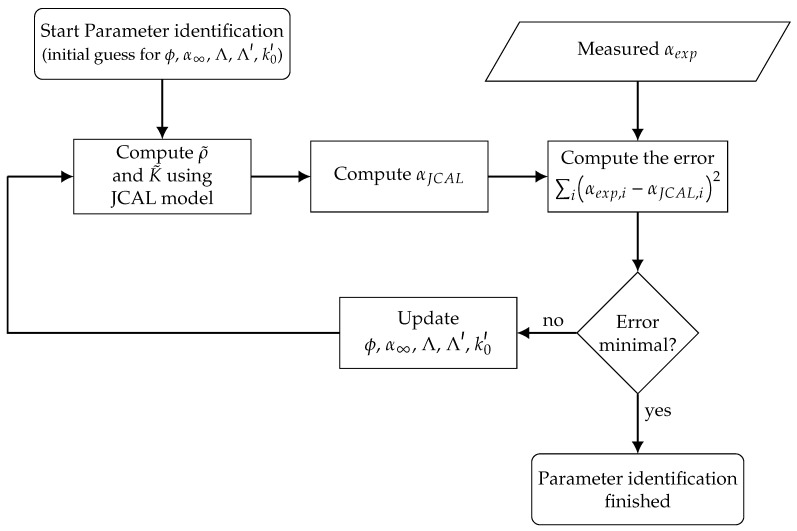
Flowchart of the inverse parameter identification process. Starting with an initial guess for the Biot parameters, the absorption coefficient is computed using the JCAL model and the results is compared to the measured data. Based on the error measure, the Biot parameters are updated until convergence is reached.

Using the Equations (1)–(4), (A1) and (A2), the absorption coefficient of a material can be computed for a given set of the six Biot parameters used for the JCAL model. Based on these formulations, an inverse parameter identification procedure is set up using an evolutional algorithm (settings: see Table A2). The updating process is sketched in Figure 4.

As described before, the flow resistivity is determined experimentally, the remaining five parameters (ϕ, $\alpha_{\infty }$, Λ, $\Lambda^{\prime }$, $k_{0}^{\prime }$) are inversely identified. Therefore, the absorption coefficient is computed ($\alpha_{JCAL}$) for a first initial guess of the parameter set. The error of the computed absorption coefficient $\alpha_{JCAL}$ and measured absorption coefficient $\alpha_{exp}$ is determined using the sum of the squared differences for all frequencies *i*:

(5) $$err=\sum_{i}{\left(\alpha_{exp,i}-\alpha_{JCAL,i}\right)}^{2}\;.$$

Based on the error measure given in Equation (5), the parameter set is updated using an evolutional algorithm as described in [66]. The objective function to be minimized yields the error measure given in Equation (5). The implementation of the evolutional algorithm is taken from the python-library scipy [67], the specific configuration is shown in Table A2 in the Appendix C.

**Figure A2:**
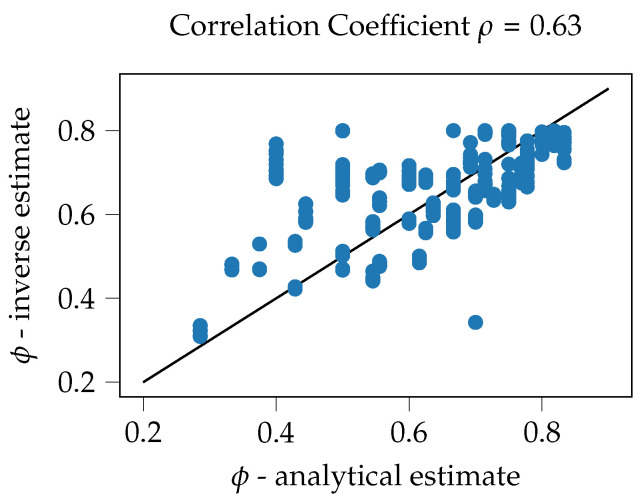
A correlation plot for the inversely estimated porosity of the specimen (vertical axis) and an analytical porosity estimate, computed using s/(s+d). A general correlation (correlation coefficient ρ=0.63) can be found. Therefore, it is assumed that the general procedure can be trusted.

An evolutional algorithm is a stochastic approach, hence several runs of the algorithm will yield somewhat different results, even though the initial parameter set remains unchanged. This can be due to different minimal solutions that are found. However, even if the same minimum is found in different runs, the stochastic nature will produce different results within small limits. This behavior is used here to enlarge the database (data augmentation) of the manufactured 50 specimens. The inverse parameter identification procedure is run ten times for each specimen, hence the database that can be used for training the ML models comprises 500 data sets with slightly different geometry-Biot parameter combinations. This behavior is due to the fact that the inverse parameter identification problem is ill-posed. Therefore, without additional information, it cannot be distinguished between the different results and whether one or the other outcome is the physically correct result and hence, all available results are used. In Figure A2, in the Appendix C, a correlation plot can be found. It shows the inversely estimated porosity of the samples over an analytical estimate that is computed with

(6) $$\phi_{analyt}=\frac{s}{s+d}\;.$$

**Figure A3:**
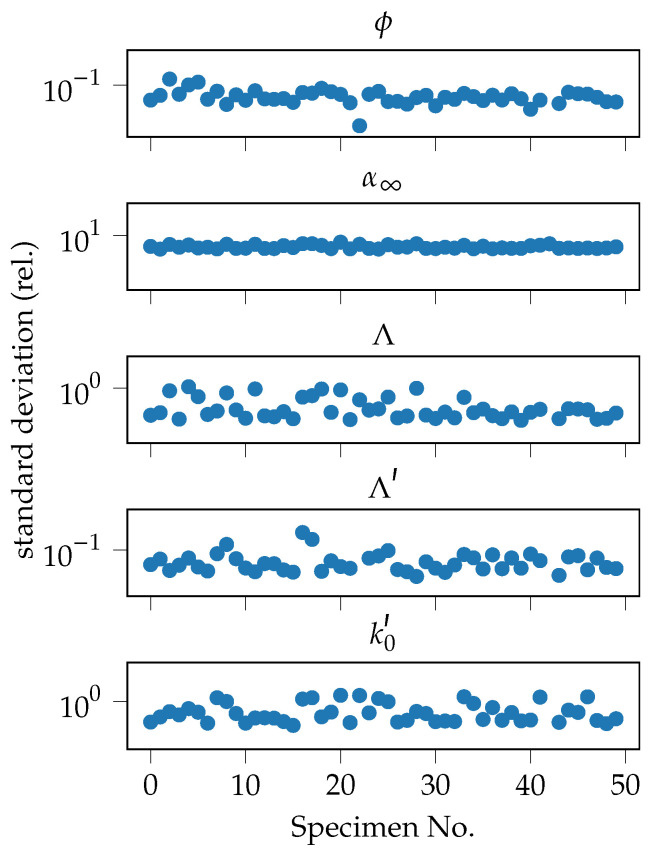
Relative standard deviation for the data augmentation procedure. The standard deviation is computed for all ten runs of the inverse parameter estimates for each specimen and normalized by the mean of the ten runs. It can be seen, that the data varies about approx. 10% for most Biot parameters.

It can be seen, that a general correlation (correlation coefficient ρ=0.63) of the inversely identified parameter and the analytical estimate can be found. The occurring differences thereby could be attributed to several reasons, the rough analytical estimate, manufacturing imperfections as well as the inverse parameter identification procedure. Therefore, in general, the procedure is trusted. In order to further investigate the data augmentation procedure, the standard deviation of the ten parameter identification runs is computed for each specimen and Biot parameter. The results can be seen in Figure A3 in the appendix (Appendix C). The standard deviation is normalized with the mean of the identified parameters. It can be seen that the variations of most parameters have a magnitude of approx. 1 × 10^−1^. Therefore, it is assumed that indeed some differences occur for each run but since the deviations are rather small, the general behavior is kept. Thus, it is assumed that the data augmentation yields meaningful results to enrich the database and the choice of input parameters is reasonable.

### 2.6. Machine-Learning for the Geometry-Biot Parameter Relations (Step 5)

**Figure 5:**
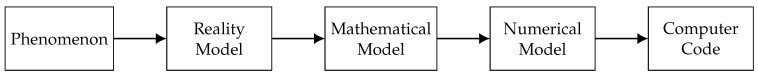
Classical model building process (from [70]).

Since Machine-Learning is a vast and still rapidly growing field, hereafter only a very short sketch of the idea and its application for engineering tasks is given. For further information, it can be referred to various textbooks, such as [68,69]. To properly explain the ML approach, it might be handy to first recall the classical model building process, as sketched in Figure 5 and described in, for example [70]:

This classical approach starts with an event or phenomenon happening in reality that is observed and, on a first level, is transformed into a so-called reality model. This reality-model is then described using mathematical equations, yielding the mathematical model which, in turn, is solved—often using numerical methods that lead to the numerical model—by means of a computer. Within this classical approach, the basic relation, which is “what the model should do” is coded within the mathematical equations of the mathematical model. These mathematical equations are themselves based on the laws of physics (or any other discipline). The basic idea of ML is, in contrast to this classical model building approach, that the relations between the model parameters are not described based on physical laws and mathematical equations, but that the relations are derived from existing data. This process of deriving the relations from data often is referred to as learning or training the ML model. Thereby, it is of tremendous importance that the desired relations are already “coded” within the existing data. The field of ML models can generally be divided into the sub-classes of supervised and unsupervised learning models. Thereby, supervised learning employs both, the input data for the model and so-called labels, which is the desired output of the model. On the other side, unsupervised learning models require the input data only and attempt to find some sort of structure within this data, for example, clustering.

Within the scope of this work, two different types of ML models are employed: (a) a K-Nearest Neighbor (KNN) model, see Section 2.6.2, and (b) an Artificial Neural Network (ANN) model, see Section 2.6.3. Both models belong to the class of supervised learning models and are applied here as such, whereas both models can be used for unsupervised learning as well. The reason for choosing these two different model types here is to allow for a comparison of different approaches regarding the necessary effort for the model training and the ability to predict a suitable set of geometry parameters for the AM process. The models differ significantly regarding the required computational effort for the training (few seconds for the KNN model, ≈25 min for the ANN model). Moreover, it is assumed that the models show a different behavior regarding the ability to inter-/extrapolates from the learned data, which is assumed to be crucial for a reasonable prediction of the Biot parameters for new specimen geometries. Such differences were already observed in the past on other problem types, therefore these models were applied here as well. Other ML model types have not been tested yet but are subject to future studies. The models are implemented using the python-library scikit-learn [71].

#### 2.6.1. Scaling of the Input and Output Data

The input data for the ML models are geometry parameters and, for the bar width, the bar spacing and the bar height measured in mm. The plane angle in measured in ${}^{\circ }$. Except from the plane angle, the magnitude of the input data is approximately 1 × 10^−1^, the magnitude of the plane angle is in the order of 1 × 10^1^. The input data is scaled using mean and standard deviation of the data with:

(7) $$x^{★}=\frac{x-\bar{x}}{y}\;.$$

In Equation (7), the quantity $x^{★}$ represents the scaled input data, $\bar{x}$ is the mean and *y* represents the standard deviation of the input data.

The Biot parameters, which is the output data of the ML models, have very different dimensions and scales, for example, the viscous and thermal characteristic lengths are geometric lengths, the static thermal permeability has the dimension of a surface, the porosity and tortuosity are dimensionless and the flow resistivity has the dimension $\mathrm{Pa}\;s/m^{2}$. Furthermore, the magnitude of these parameters is rather different and comprises a large range of values; for example, the magnitude to the flow resistivity is 1 × 10^3^–1 × 10^5^, the magnitude of the static thermal permeability is approx. 1 × 10^−8^. It could be found that the training process becomes rather unsuccessful when the input and output data is used directly. This is expected to be a result of the large range of values within the data. Therefore, variable scaling is introduced and applied to the Biot parameters in order to result in training data comprising a smaller value range. The resulting scaled Biot parameters are shown in Table 3:

The flow resistivity remains unscaled. This scaling is assumed to only improved the training performance of the ML models but does not affect the general procedure.

#### 2.6.2. K-Nearest Neighbors

K-Nearest Neighbor (KNN) ML models are a very popular approach and have been successfully applied to classification problems. For further information on the topic, the reader is referred to the the vast amount of textbooks and papers on this topic, such as [69,72,73,74]. For the classification, the labels of the existing data serve as the classes into which new data instances shall be classified during the application of the model. As inputs, KNN models use a feature vector for each data instance. Now a class is formed by all input data instances, whose feature vectors are similar to each other. During the application of the model, new data instances with their individual feature vectors are compared to the feature vectors of the classes the model initially had learned and the new instance is classified into that class to which its own feature vector is most similar. The similarity thereby is measured using certain distance measures that are specified by the user. Another parameter that determines the behavior of the model is the number of neighbors (*k*) that are accounted for during the classification process. Due to expected noise within the data, it is common to compare new data instances to more than one instance. The parameter *k* determines the number of data sets to which the new data instance is compared. The distance measure and the number of neighbors are the so-called hyperparameters of the ML model. The appropriate choice of these hyperparameters is crucial for a successful application of ML models. KNN models can be applied to regression problems as well. In such cases, the output quantity is not a single and discrete class but is computed as a continuous quantity from the *k* neighbors [69].

**Figure A4:**
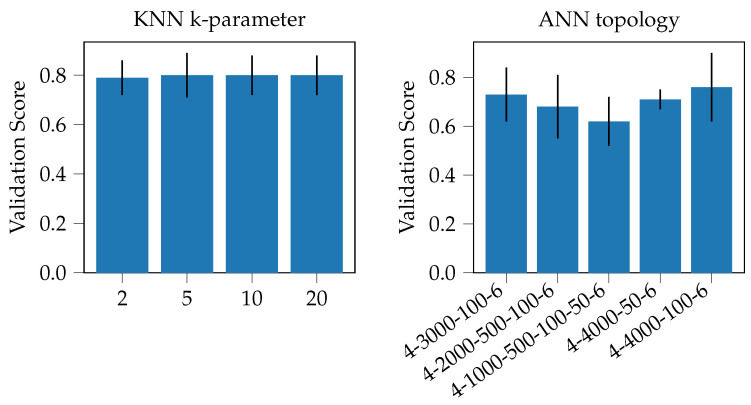
Hyperparameter tuning of the KNN and ANN model. The tuning is done on the validation data during the 3-fold cross-validation. The error bars indicate the 95% confidence interval of the variation during the cross-validation procedure.

The KNN model employed within this work uses a *k*-parameter of k=5 and weights the different neighbors of the new data instance based on the inverse of its distance to the learned data instance. The choice of the *k*-parameter results from the hyperparameter tuning that was done on the validation data set. In Figure A4, the results of the cross-validation with different *k*-parameters are shown. It can be seen, that the cross-validation score is improved from k=2 to k=5 but even higher values for *k* do not further improve the score. Therefore, k=5 is chosen. The validation and test scores for the KNN model can be found in Table 4. The model is used as a multi-output regression model.

#### 2.6.3. Artificial Neural Network

The approach of the Artificial Neural Networks is based on the idea of the so-called perceptron [75,76]. A perceptron can be understood as a unit that has input channels taking a data vector and yielding a certain scalar output based on the input data vector. Therefore, a weighted sum of the input vector elements is computed and the result of this sum serves as the argument to the so-called activation function. This activation function then computes the perceptron’s output and can yield both, discrete or continuous values. The most simple category of ANNs are multi-layer perceptrons, for which a multitude of single perceptrons (or neurons) are organized in several layers and interconnected to each other, so the output of one neuron is the input to one or more subsequent neurons in the following layer. This connection between the layers is further associated with a certain weight, the weights of each connection within the ANN is adjusted during the training process. Therefore, several optimization algorithms can be used. This is necessary since the weight adjustment problem is ill-posed. The main hyperparameters of the ANN thus are the topology (number of neurons/layer and number of layers), the activation function(s) and the optimization algorithm. One of the main advantages of multi-layer perceptron neural networks over single perceptrons is the ability to learn and reproduce non-linear functional relations. Indeed, much more complex versions of this multi-layer perceptron prototype exist today, for example, recurrent neural networks, convolutional neural networks and many more [69,77].

**Figure 6:**
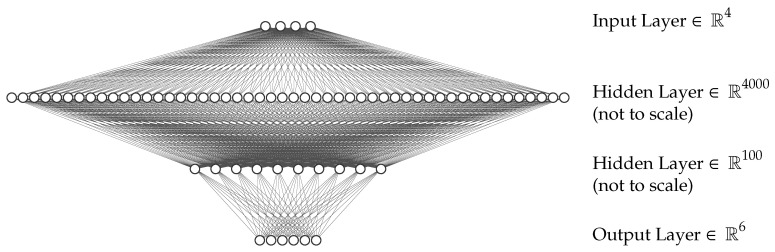
Sketch of the ANN used within this work, drawn using [78]. The input layer has four neurons and takes the geometry parameters *w*, *s*, *h*, φ of the specimen. The output layer has six neurons for the Biot parameters Ξ, ϕ, $\alpha_{\infty }$, Λ, $\Lambda^{\prime }$ and $k_{0}^{\prime }$. The two hidden layers have 4000 and 100 neurons (not to scale), respectively. The activation function for all neurons is the ReLU function.

The ANN employed within this work is set up as a four-layer feed-forward ANN with two hidden layers. The input and output layers have 4 and 6 neurons, respectively, according to four geometry parameters that serve as inputs and the six Biot parameters Ξ, ϕ, $\alpha_{\infty }$, Λ, $\Lambda^{\prime }$ and $k_{0}^{\prime }$ as outputs. The hidden layers employ 4000 and 100 neurons, respectively. The activation function used for all neurons is the so-called Rectified Linear Unit (ReLU), the optimization algorithm for adjusting the weights is the Adam algorithm. The resulting ANN model is sketched in Figure 6.

The hyperparameters (the number of layers, neurons, activation function and optimization algorithm) are derived during a 3-fold cross-validation procedure. Thereby, the topology mentioned above (4-4000-100-6) reached the best validation score, as shown in Figure A4. The ReLU activation function and the Adam optimization algorithm were the only choices with which a converging training process could be reached. The validation and test scores for the ANN model can be found in Table 4.

## 3. Results and Discussion

Within this section, the obtained results of the work are presented. In the first part (Section 3.1), the manufactured specimen are investigated regarding their actual geometry and their acoustic parameters (Steps 1–3 of Figure 1). Therefore, in Section 3.1.1, an optical inspection of two specimens is presented. These specimens are found to show the highest and lowest mean absorption coefficient over the entire frequency range and thus represent some kind of extreme values. The acoustic inspection is presented in Section 3.1.2. Here, the measured flow resistivity and absorption coefficient are presented. The results of the inverse parameter identification procedure are shown in Section 3.1.3 (Step 4). The second part (Section 3.2) of this section is devoted to the results of building the ML models (Step 5). Thereby, the results of the training processes are shown and the accuracy of the predicted Biot parameters is assessed for two specific samples. The obtained ML models are applied in Section 3.3 for the design of new absorber specimens that are intended to show a specific prescribed absorption coefficient over frequency (Step 6).

The overall idea of the procedure within this work is to generate models that connect the specimen geometry to the Biot parameters. The reason for choosing the Biot parameters (which are material-specific parameters) rather than directly predicting the behavior of the material, for example, the absorption coefficient, is that the Biot parameters can be used for subsequent analyses and other contexts. This is addressed in Section 3.4 by manufacturing a new specimen with a varied specimen height *l* and prediction of the absorption coefficient for a broader frequency range. Height and frequency range were kept constant for the initial specimen population with which the Biot parameters are identified. Now the Biot parameters that were obtained by the ML models are used to predict the absorption coefficient of a specimen with a larger specimen height and for a broader frequency range. This shows, that the obtained models are suitable as well to design porous absorbers with a different geometry.

### 3.1. Inspection of the Specimen Population

Within this section, the results of the inspection of the initially manufactured specimen population are shown. Thereby, in Section 3.1.1 the results of a first optical inspection are presented and the generally correct AM process is verified. These results (the specimen micro-scale geometry) serve as inputs (features) to the ML models. In Section 3.1.2, the results of the acoustic measurements are presented, which are the measurements of the flow resistivity and the absorption coefficient. In Section 3.1.3, the results of the inverse identification of the Biot parameters are shown. These Biot parameters serve as outputs (labels) for the ML models, whose prediction performance is presented and assessed in Section 3.2.

#### 3.1.1. Results of the Optical Investigation of the Additively Manufactured Specimens

**Figure 7:**
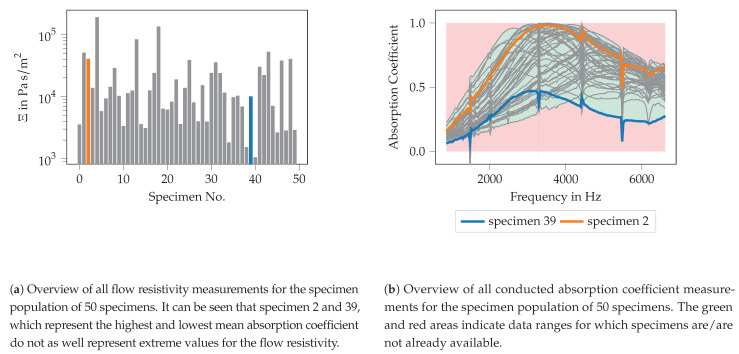
Measured data (flow resistivity and absorption coefficient) of all 50 investigated specimens. The specimens 2 and 39 are marked in orange and blue, respectively, and represent those specimens with the highest and lowest mean absorption coefficient in the frequency range.

To assess the manufacturing accuracy of the additively manufactured test specimens, an actual-target comparison of the initial geometry and the manufactured test specimen was carried out. Specimens 2 and 39 are selected as examples since these specimens show the highest and lowest mean absorption coefficient over the frequency range of interest (see Figure 7b) and thus provide some kind of extreme values.

**Figure 8:**
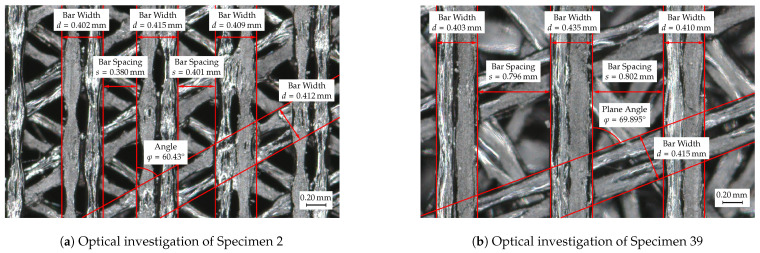
Actual-target comparison of specimens 2 and 39 using Keyence VR5200 3D surface profilometer. (**a**) shows specimen 2 with only slight deviations in the actual-target comparison. However, some small tapers can be seen in the filament strands. (**b**) shows specimen 39, which also shows only minimal deviations in the actual-target comparison.

Test specimens 2 and 39 are generated using the variation parameters given in Table 5 using OpenSCAD [59] and should have the specified values after additive manufacturing. Manufacturing accuracy is assessed through the use of optical metrology. Specifically, a Keyence VR5200 (Neu-Isenburg, Germany) 3D surface profilometer with microlens was used. Figure 8 shows the test specimens 2 (Figure 8a) and 39 (Figure 8b) at 80× magnification.

Test specimen 2 shows a relatively uniform production pattern, with a partial tapering of the filament strands in the spaces between the underlying bars. Two applied filament strands each form the individual webs of the acoustically effective structures, the width of which is 0.40 mm for test specimen 2. Evaluation through optical measurement technology showed that the resulting width of the bars lies within a range of 0.402–0.415 mm, which corresponds to a maximum deviation of roughly 4%. In addition to the bar width, the bar spacing was also examined, which should also be 0.40 mm. In contrast to the bar width, a small variation of the values can also be measured here. The resulting bar spacing have a value between 0.38 and 0.401 mm, which corresponds to a maximum deviation of roughly 5%. The resulting plane angle between the individual bar structures should have a value of 60${}^{\circ }$, which was achieved with a value of 60.43${}^{\circ }$ with very small deviations (< 1%).

A similar manufacturing analysis was also carried out for test specimen 39, see Figure 8b, by also determining the bar width, bar spacing and plane angle. In contrast, to test specimen 2, the applied filament strands are much more homogeneous in their width and only minor tapers in the bar width can be measured. At maximum, a bar width of 0.403–0.435 mm could be determined, which corresponds to a maximum deviation of roughly 9%. However, the vast majority of the bars show a much smaller deviation from the required 0.40 mm. The bar spacing of the test specimen 39 has a value between 0.796 and 0.802 mm, which corresponds to a maximum deviation of < 1 of the required 0.8 mm. The defined plane angle of 70 ${}^{\circ }$ was also satisfactorily achieved with a value of 69.895${}^{\circ }$.

All in all, the manufacturing accuracy can be classified as acceptable and only slightly fails to meet the desired dimensions. The highest measured deviation is at a bar width of 0.435 mm and thus corresponds to a deviation of 0.035 mm. However, the vast majority of the tested bars show significantly smaller deviations. Test specimen 2 also exhibits only minor deviations from the desired geometry. Clarification is needed for the partial tapering of the filament strands in Specimen 2, which may be due to insufficient thermal energy or slow cooling of the strands, resulting in elongation of the filament strands and partial tapering.

#### 3.1.2. Results of Acoustic Measurements of the Specimen Population

The results of the measurement of the flow resistivity are shown for all 50 specimens in Figure 7a. It can be seen, that flow resistivity values in the range of 1535.90–51,061.20 Pa s/m${}^{2}$ are observed. Specimen 2 and specimen 39 are marked in orange and blue, respectively.

These specimen exhibits the largest (specimen 2) and lowest (specimen 39) mean absorption coefficient over the frequency range, as shown in Figure 7b. Nevertheless, the flow resistivity alone does not seem to explain the acoustic behavior since other specimen with even higher (for example specimen 5) and lower (specimen 41) flow resistivities exist whose mean absorption coefficient lies between the ones of specimen 2 and specimen 39.

In Figure 7b, all absorption coefficient curves of all specimens are plotted over frequency. In this figure, no distinction between the different specimens is made, only the curves of specimen 2 and specimen 39 are highlighted. These curves are the curves with the highest/lowest mean absorption over the entire frequency range. All other absorption curves show a mean absorption coefficient between these two highlighted curves, however, the spectral distribution might change. Furthermore, a red and a green area is marked. These areas indicate where no data is obtained from the entire specimen population. They become relevant for the discussion of the performance of the ML-models for the absorber design in Section 3.3. For the majority of the specimens, an absorption coefficient curve with a distinct maximum can be seen. This maximum can be found in a frequency range between approx. 2800 and 4200 Hz. Classical porous materials like foams are expected to show a rather broad and flat course of the absorption coefficient for high frequencies. Nevertheless, the absorption maximum for the specimen used here could already be flattened in comparison to former studies, in the sense that the frequency range with high absorption characteristics gets broader. These measured absorption curves are used for the inverse parameter identification procedure described in Section 2.5 for defining the target function.

The absorption coefficient curves shown in Figure 7b show some “spikes” or “kinks” at specific frequency points. The reason for this behavior could not be revealed by now. Since all “spikes” are located at the same frequencies and occur for all measured specimens, it is assumed that the “spikes” result from a systematic error, probably in the transfer function computation of the applied two microphone method. Within this procedure, the measured transfer function is corrected for the microphone phase mismatch using a transfer function measured prior to the measurement campaign. This correction procedure might be ill-conditioned for the distinct frequency points. Therefore, the results away from the spikes are supposed to be trustworthy.

#### 3.1.3. Results of the Inverse Parameter Identification

**Figure 9:**
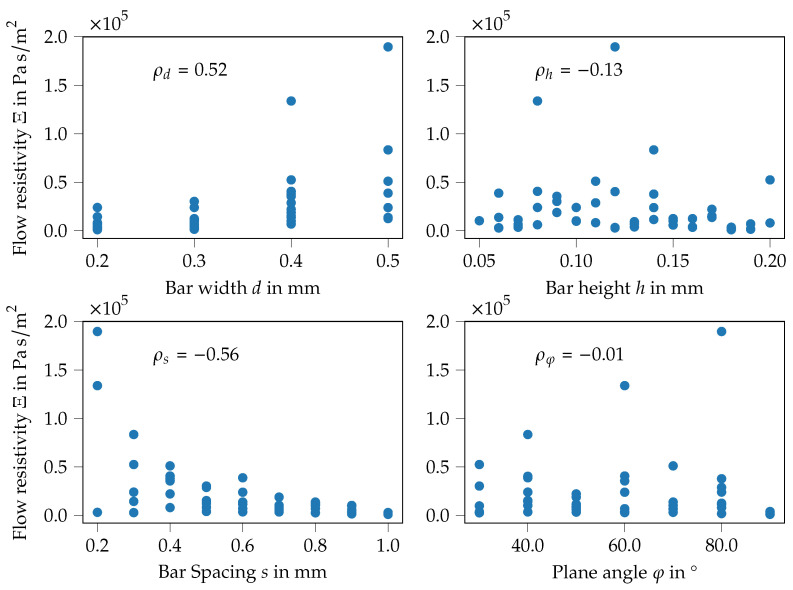
Correlation of the flow resistivity and the design variables of all manufactured specimens. It can be seen that only the bar width and the bar spacing show a relevant correlation with the flow resistivity, whereas the flow resistivity does not (linearly) depend on the bar height and plane angle.

Based on the measured flow resistivity and absorption coefficient, the Biot parameters are inversely derived using the procedure described in Appendix C. In order to give a first insight into these data, a correlation analysis between the flow resistivity Ξ and the design variables is conducted. The variation of the design parameters of the specimen are expected to change the flow resistivity, therefore the plot in Figure 9 shows the flow resistivity of all specimens over the geometry parameters, these are the bar width (top left), the bar height (top right), the bar spacing (bottom left) and the plane angle (bottom right). Moreover, the correlation coefficient ρ (throughtout the manuscript, Pearson’s correlation coefficient $\rho =\frac{cov\left(X,Y\right)}{\sigma_{X}\sigma_{Y}}$ is used. Here, both the flow resistivity and the geometric quantities are assumed to be the random variables *X* and *Y*, respectively) for the two quantities are computed and given in the plots.

For the bar width, (see Figure 9, top left) the stages of the LHS sampling strategy can be seen, values from 0.20 to 0.50 mm with a step width of 0.10 mm are used. Thereby, the flow resistivity generally increases with the bar width, resulting in an intermediate positive correlation of $\rho_{d}=0.52$. A correlation of similar magnitude, but in the opposite direction can be detected for the bar spacing, see Figure 9, bottom left, with $\rho_{s}=-0.56$. Here the flow resistivity decreases with increasing bar spacing. Both correlations of the bar width and the bar spacing to the flow resistivity seem reasonable—increasing the bar width decreases the open channel area, increasing the bar spacing enlarges the open area. On the other side, the bar height (see Figure 9, top right) and the plane angle (see Figure 9, bottom right) show only a very weak correlation to the flow resistivity. In total, the plots show that no trivial dependency can be found to adjust the specimen dimensions properly in order to tailor their absorption characteristics. This finding further motivates the application of ML-models to design absorbing structures.

### 3.2. Setup of Machine-Learning Models for Predicting Biot Parameters from the Specimen Geometry

The training process for both ML models employed within this work is composed as follows. The 500 available data sets generated from the genetic algorithm during the inverse parameter identification are split into two sub-sets, a training and validation data set (A) and a test data set (B). The test data set comprises 20% (100 data instances) of the available data and is used for the performance test at the end of the training process, only. The test data set is chosen randomly from the available 500 data instances. The hyperparameters, namely the number of nearest neighbors and the distance measure of the KNN model and the number of layers/neurons and activation function of the ANN model, were found using a three-fold cross-validation schema [69]. Therefore, the remaining 80% (data set A, comprising 400 data instances) of the available data is split into three parts of equal size (133 data instances), from which two (266 data instances) are used for training the model (training data set) and the third for evaluating its performance (validation data set). This procedure is run three times, so all elements of the data set A have been used at least once for training and testing, respectively. The performance is measured in terms of the cross-validation score, this is the mean of all scores during the cross-validation. The cross-validation score, as well as the test score, are measured with the so-called coefficient of determination $R^{2}$ [79]. This quantity measures the amount of variance explained by the model and thus is often used for measuring the prediction accuracy of dependent variables from independent variables [80]. The $R^{2}$ measure normally varies between 0 and 1, whereas $R^{2}=1$ refers to a perfect prediction by the model, $R^{2}=0$ refers to a model always predicting the mean of the data. Under specific conditions, values of $R^{2}$ below 0 or above 1 can be obtained as well.

The cross-validation procedure is run several times with varying hyperparameter in order to find parameters that perform well (highest $R^{2}$-score) for the given problem. Figure A4 shows the results of this hyperparameter tuning for both, the KNN and the ANN model, respectively. It can be seen, that for the KNN model a *k*-parameter of larger than k=5 does not lead to a further improvement of the results, therefore k=5 is chosen. For the ANN model, different topologies were investigated with at least two and a maximum of four hidden layers. From Figure A4 it can be seen, that the model with the 4-4000-100-6 topology performed best, therefore this model is used for all investigations. The hyperparameters activation function (ReLU) and optimization algorithm (Adam) were the only ones with which a converging solution could be obtained. Therefore, these are used as well.

After fixing the ML model design using the chosen hyperparameters mentioned above, the models are tested using the remaining share of the data for estimating their performance with regard to yet unseen data. This performance is measured using the test score. The resulting scores are summarized for both models in Table 4.

From Table 4 it can be seen, that both models reach training and test scores above 0.70 whereas the KNN model performs better during the cross-validation with a higher score and lower uncertainty. Nevertheless, the ANN model performs better on the unseen test data. This may be due to the fact that before the test procedure, both models have been trained with all data sets from the cross-validation, thus more information was available to train the model. Another effect might be that it is suspected that ANN models have a better ability to extrapolate from know to unseen data than other models. Furthermore, it should be noted that the KNN model requires only a few seconds of computing time on an intermediate laptop for the training process, whereas the ANN model requires a computation time of approx. 25 min. To summarizing, the models both show a reasonable performance in terms of prediction accuracy whereas the required computational effort for the training is very different. Nevertheless, since for the purpose of this work the focus is on the prediction capability rather than computational effort, both models seem to be reasonably applied to the absorber design procedure.

#### 3.2.1. Performance of the KNN Model for Predicting Biot Parameters

**Figure 10:**
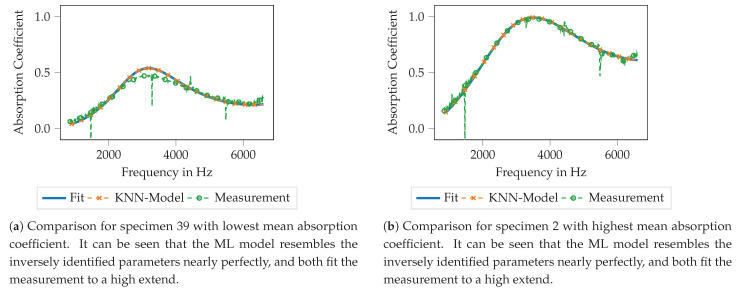
Comparison of the measured absorption coefficient, the absorption coefficient computed from the inversely identified Biot parameters (’Fit’) and absorption coefficient computed using the Biot parameters outputted by the KNN model.

In Figure 10, the performance of the KNN model is illustrated. Therefore, the blue line shows the absorption coefficient that is computed using the Equations (1)–(4), (A1) and (A2) with the inversely identified Biot parameters. The orange line indicates the computed absorption coefficient using the mentioned set of equations if the Biot parameters computed using the KNN model are used. For comparison, the green line shows the measured absorption coefficient. The plot Figure 10a shows the results for specimen 39, the specimen with the lowest mean absorption coefficient. It can be seen that the Biot parameters, computed using the ML model (orange), practically perfectly approximate the inversely identified parameters (solid line) with regard to the subsequently computed absorption coefficient. Both approaches match the measured data to a reasonable extend, nevertheless some deviations can be observed around the maximum of the absorption coefficient at approx. 3600 Hz.

Similar findings can be observed for the specimen with the highest mean absorption coefficient, specimen 2, see Figure 10b. For this specimen, all shown curves match nearly perfectly. Hence, the inversely identified Biot parameters resemble a good approximation of the measured absorber and the trained ML model is able to reproduce the Biot parameters from the geometry pretty well.

**Figure 11:**
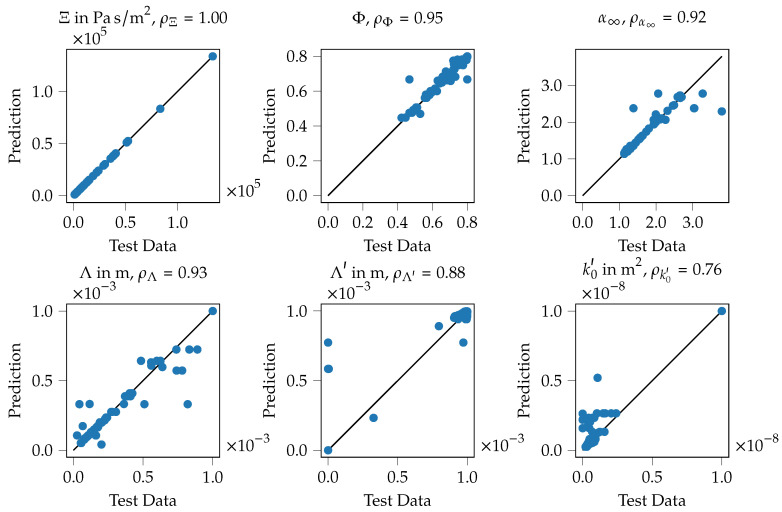
Correlation graphs for inversely identified Biot parameters and their corresponding prediction by the KNN model. It can be seen that all quantities are predicted with reasonable accuracy, thus the model is qualified for further application.

An overview of the performance of the KNN model over the entire training data is given in Figure 11. The six shown plots refer to the six parameters of the JCAL model, for each plot the test data is plotted in the horizontal axis, the prediction of the model is plotted in the vertical direction. A perfect training result would produce only datum points on the solid black line, the prediction would fit the test data perfectly. For each quantity, Pearson’s correlation coefficient ρ is given in the plot’s title. It can be seen that for the flow resistivity, see Figure 11 top left, a practically perfect fit between the test data and the ML model can be obtained. For the porosity (top middle), the tortuosity (top right) and the viscous characteristic length (bottom left) correlation coefficients above 0.90 can be obtained. Nevertheless, some outliers can be observed as well, for example for the thermal characteristic length (bottom middle), two data points show a value of approximately 0.00 m for the test data, but are predicted with values of $0.55\times {\mathrm{10}}^{-3}$ m and $0.70\times {\mathrm{10}}^{-3}$ m, respectively. It is expected, that such outliers occur when for these specific specimens the combination of the different geometry parameters has a negligible influence on the acoustic parameters. This rather low influence results in a low potential to extract information about the relation between the parameters and thus results in wrong training results. In total, the predictions of the KNN model are in good agreement with the input data, hence the training process is assumed to be successful.

#### 3.2.2. Performance of the ANN Model for Predicting Biot Parameters

**Figure 12:**
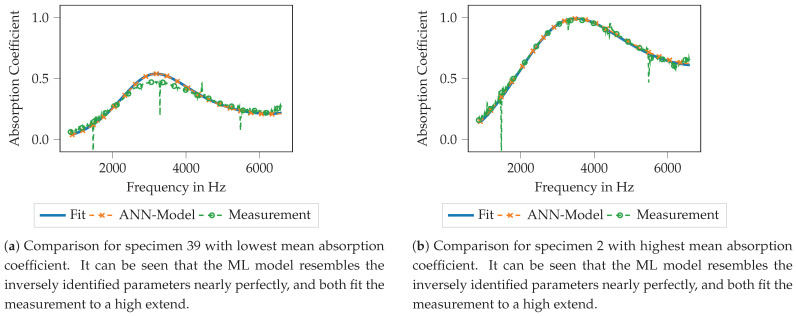
Comparison of the measured absorption coefficient, the absorption coefficient computed from the inversely identified Biot parameters (’Fit’) and absorption coefficient computed using the Biot parameters outputted by the ANN model.

Very similar observations can be made for the ANN model, as shown in Figure 12. For specimen 39 with the lowest mean absorption coefficient (see Figure 12a), the computed absorption coefficient with the Biot parameters of the ML model output fits nearly perfect the absorption coefficient computed using the inversely identified Biot parameters. Moreover, both approaches match the measured data with reasonable agreement. Here as well, the largest deviations can be found around the maximum of the absorption coefficient. For the specimen no. 2 (see Figure 12b), the findings are pretty similar. Only in the very high-frequency regime, above 6000 Hz very small deviations between the used models can be observed, still, both models match the measured data very well. Summarizing, the inverse parameter identification process as well as the subsequent training of the ML models seems to result in data models that are capable of resembling the original data and thus the printed porous absorbers reasonably well.

**Figure 13:**
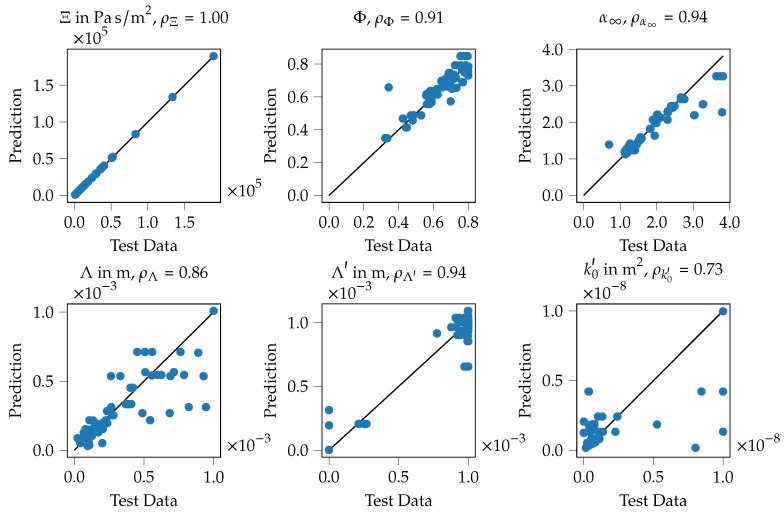
Correlation graphs for inversely identified Biot parameters and their corresponding prediction by the ANN model. It can be seen that all quantities are predicted with reasonable accuracy, thus the model is qualified for further application.

For the ANN model as well the correlation of the input data and the predictions are shown for all Biot parameters in Figure 13. The findings are similar to the KNN model; the flow resistivity shows a practically perfect fit of the test data and the model’s prediction, porosity, tortuosity and the thermal characteristic length show values of the correlation above 0.90. As well, some outliers can be observed, for example for the static thermal permeability (bottom right). Here, some test data points with rather large values of 0.80 × 10^−8^ –1.00 × 10^−8^ m^2^ can be seen that are predicted by the ANN model with rather low values of 0.01 × 10^−8^–0.45 × 10^−8^ m^2^. Here as well, it is assumed that the geometry parameter combination has only a low effect on this acoustic parameter which leads to rather inaccurate predictions. To summarizing, the training of the ANN model can be assumed to be successful here as well.

### 3.3. Application of the Machine-Learning Models for Absorber Design (Step 6)

**Figure 14:**
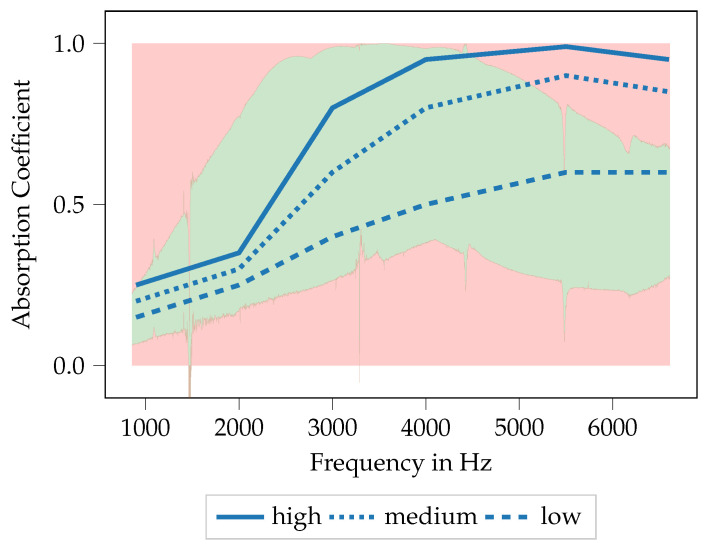
Target curves for the porous absorber design. Red and green areas mark known/unknown regions that are/are not covered by the existing specimen yet. The target curve “low” lies completely within the known data range, it is assumed that here only interpolation within the available data is required. The target curves “medium” and “high” leave the known range above approx. 5000 Hz, here the ability to extrapolate from the known data is required.

To now generate porous absorbers that show predefined absorption characteristics, three target curves are defined. These three curves are referred to as low, medium and high and are shown in Figure 14. The course of the different target curves is chosen arbitrarily, nevertheless, they are intended to resemble common application cases. The idea is to generate printable absorber designs using the ML models described in the previous section and to evaluate to which extend the printed absorbers show the desired characteristics.

In Figure 14, the target curves for the absorption coefficient are plotted over frequency, thereby the frequency range is kept identical to the specimen already available. All target curves start at rather low absorption coefficients at low frequencies and show an increase of the absorption coefficient with frequency. The curves thus resemble a general characteristic of porous absorbers. Nevertheless, when compared to the absorption characteristics shown in Figure 7b, it should be noted that the frequency range with a high absorption coefficient is generally broader than it is with the available specimen. Furthermore, the red and green areas from Figure 7b are shown again here, indicating the areas for which suitable specimen designs are already available. It should be noted here that the curve low remains within the green area for all frequencies, whereas the curves medium and high leave the green area for frequencies above approx. 5200 Hz and 4300 Hz, respectively. This will be important for the result discussion in Section 3.3.1 and Section 3.3.2, respectively. Hence, to generate specimens whose absorption coefficient follows these curves, an extrapolation capability of the ML models is required.

**Figure 15:**
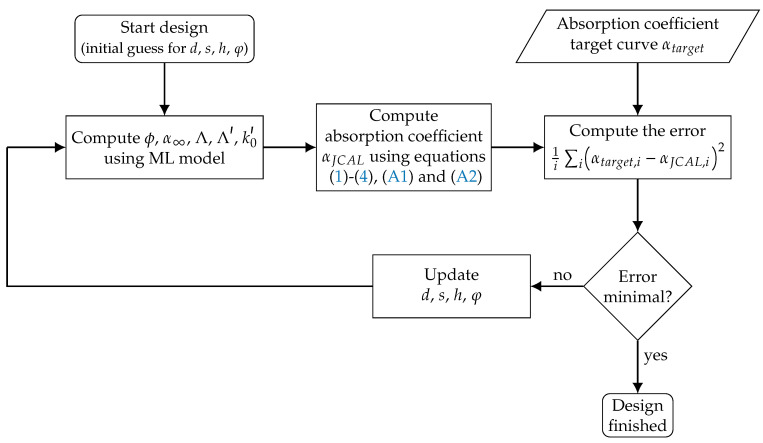
Flow chart of the inverse absorber design process (Step 6 from the flowchart in Figure 1). Based on an initial guess of the design parameters, the corresponding Biot parameters and the resulting absorption coefficient is computed. Based on the given error measure with respect to the target, the design parameters are updated until convergence is reached.

The approach to design porous absorbers using the trained ML models is as follows. As the target value of the absorption coefficient is given by the target curves and the required Biot parameters are unknown, an inverse procedure is established. The procedure is sketched in Figure 15. An optimization strategy similar to the inverse parameter identification procedure described in Appendix C is set up. The used evolutional algorithm (for parameters, see Table A5) is the same as in the parameter identification procedure, only a slight difference is made for the error measure. Here, the mean of the squared difference of the target curve $\alpha_{target}$ and the absorption coefficient computed using the Biot parameters from the ML model in combination with the JCAL model $a\alpha_{JCAL}$ is used:

(8) $$err=\frac{1}{i}\sum_{i}{\left(\alpha_{target,i}-\alpha_{JCAL,i}\right)}^{2}\;.$$

Starting with an initial guess for the geometry parameters, the Biot parameters are computed using the ML model and the resulting absorption coefficient is computed using Equations (1)–(4), (A1) and (A2). The results are compared to the chosen absorption coefficient target curve and the error is computed. Based on this error measure, the optimization algorithm updates the geometry parameter set. These new geometry parameters are again fed into the ML model to compute the resulting Biot parameters with whom the loop starts again until the requested error bound is reached. The optimization based on the evolutional algorithm is set up as a bounded optimization. The bounds are chosen based on the applied MEX process and given in Table 6.

After completing the material design using the process described here, the resulting designed absorbers are printed equivalently to the specimen used for the data generation. The resulting specimens are measured in the impedance tube regarding their absorption coefficient and using a 3D surface profilometer regarding their geometry. The results are presented in the following sections.

#### 3.3.1. Specimen Design with K-Nearest Neighbor Approach

The first attempt for generating printable porous absorbers that show a prescribed absorption characteristic is done using the KNN model. The design parameters for all three target curves and the corresponding measured values are shown in Table 7. In Table 7, it can be seen that the actually manufactured geometry is rather close to the design parameters. The maximum evaluated deviation is 0.04 mm.

**Figure A5:**
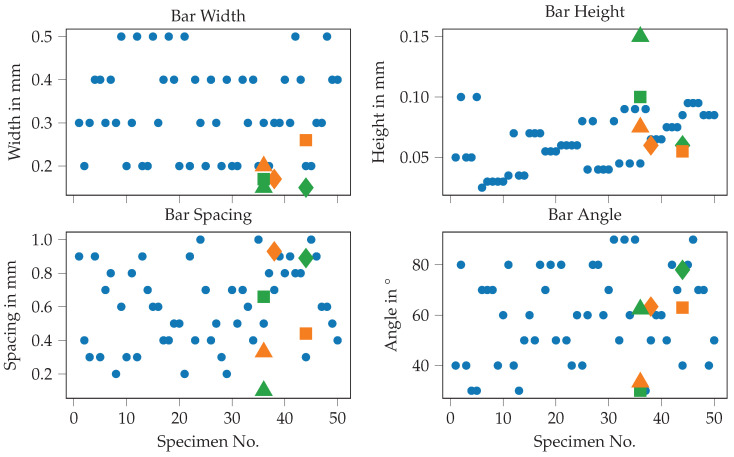
Overview over all designed Spec.s using the proposed ML approach. The colors refer to the ML model and the Spec. start population: Blue: basic population, Orange: KNN model, Green: ANN model. The symbols indicate the absorption coefficient target curve: ♦: target curve “low”, ■: target curve: “medium”, ▲: target curve: “high”.

In Figure A5, in the Appendix G, the KNN designs are shown in orange together with the geometry parameters of the initial 50 specimen population. It can be seen that all designs found by the KNN model lie within the learned data range. Indeed, the designs are new in the sense that most chosen values were not investigated and learned before. However, the data range given from the initial specimen population is not left by the KNN model.

**Figure 16:**
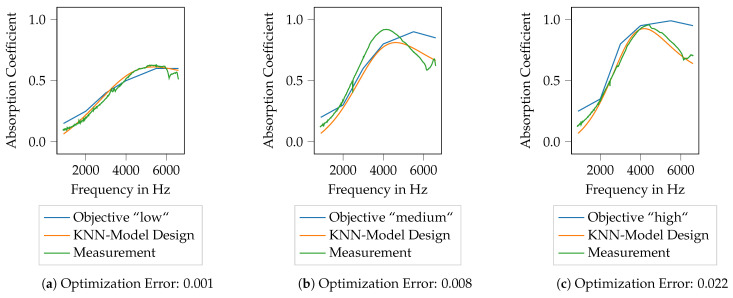
Design of porous media using an KNN machine-learning model for three different target curves. It can be seen that, especially for the targets “low” and “high” the predictions are met with high accuracy. However, the KNN model is not able to extrapolate from the know data range, as the prediction does not follow the target curves for “medium” and “high” above approx. 5000 Hz.

The results of the predicted and measured absorption coefficient are shown in Figure 16, whereas Figure 16a shows the results with the target curve “low”, Figure 16b,c show the results for the target curves “medium” and “high”, respectively. For all plots, the blue curves show the relevant target curve, the orange line shows the expected absorption coefficient of the designed material that is based on the JCAL model and the green line shows the measured data of the printed specimen. In the figure caption, the optimization error after the material design is given, this is the resulting error between the target curve and the expected absorption coefficient of the designed material as given by Equation (8). It should be noted that for computing the orange design curves, the actual manufactured parameter values from Table 7 are used.

For the target curve “low”, see Figure 16a, it can be seen that the material design using the KNN model can produce reasonable results. The optimization error after the material design is very low, the predicted absorption coefficient matches the target curve fairly well. Furthermore, the measured data shows that the absorption coefficient of the printed specimen follows the predicted absorption coefficient very well. For the case of a low absorption coefficient, the proposed procedure seems to produce reasonable results. The results for the target function “medium” as shown in Figure 16b, the results are rather different. For instance, it can be stated that the predicted absorption coefficient is able to follow the target curve only in the frequency regime below approx. 4000 Hz. Before commenting on the course of the predicted absorption coefficient, it should be noted that the measured data fit the designed material rather well. Indeed, the deviations between the measured and the predicted data are higher than for the target curve “low” but the general course is met rather well. Interesting here is the course of the predicted absorption coefficient in the higher frequency regime above 4000 Hz. Here, the predicted absorption coefficient is not able to follow the target curve anymore. This behavior is crucial and special to the KNN model. It is assumed here that the reason for this behavior is the inability of the KNN model to extrapolate from already learned information. Recalling the introduction of the target curves in Figure 14, it can be seen that the target curves “medium” and “high” leave the area of the learned data between 5200 Hz and 4300 Hz, respectively. Hence, to generate designs that exhibit an absorption coefficient beyond the learned data, the applied models must have the ability to extrapolate from the learned data to some extend. The results of the design process for the target curve “high” are shown in Figure 16c. Regarding the predicted absorption coefficient, a similar behavior as for the target curve “medium” can be observed. The predicted absorption coefficient follows the target rather well below 4000 Hz but is not able to follow for higher frequencies. Accordingly, the optimization error with a value of 0.022 is rather high. Nevertheless, the measured data show a very good agreement with the predicted absorption coefficient, hence the computed geometry parameters prove to work for practical applications.

It should be noted here that all measured absorption coefficient curves show a rather strange behavior above 6200 Hz, as the absorption coefficient curves show some sort of “kink” and increases for larger frequencies, whereas it monotonically decreases for lower frequencies. This behavior is not common for porous absorbers since such materials are expected to show a rather smooth course of the absorption coefficient over frequency. It is expected here that this “kink” is caused by an imperfect fit of the specimen within the impedance tube that leads to enclosed air-filled voids between the specimen and the tube’s wall. Such voids might serve as a high-frequency resonator, resulting in a corrupted absorption coefficient measurement. The imperfect fit of the specimen again might be due to manufacturing inaccuracies.

To summarize, the KNN model can generate printable geometry descriptions for the design of porous media. Nevertheless, it is required that the requested designs lie somehow within the previously learned data range, the ability to extrapolate outside the learned data range seems rather limited.

#### 3.3.2. Specimen Design with Artificial Neural Network Approach

The material design task using the ANN model leads to rather different designs compared to the KNN model. The predicted parameter values for all geometry parameters and target curves are shown in Figure A5 in the Appendix G. It can be seen that the ANN model generates both, geometry values inside and outside the data range provided by the specimen population. Hence, some ability to extrapolate from the learned data range can be confirmed. A comparison of the manufactured and designed geometry parameters is shown in Table 8.

It can be seen that generally similar parameter ranges as obtained with the KNN model are reached. The manufacturing accuracy as well is similar to the KNN model, the maximum deviation between the manufactured and predicted geometry parameters is 0.05 mm for the bar spacing, target curve “low”. All other (measurable) parameters are manufactured with a deviation of 0.01 mm.

**Figure 17:**
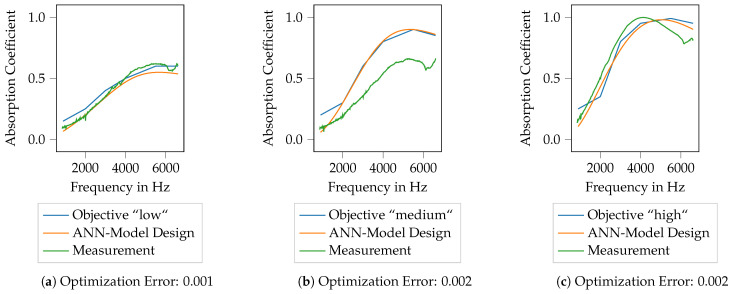
Design of porous media using an ANN machine-learning model for three different target curves. It can be seen that, especially for the targets “low” and “high” the predictions are met with reasonable accuracy. The ANN model is able to extrapolate from the know data range, as the prediction follows the target curves for “medium” and “high” above approx. 5000 Hz. The large deviations of the measurement for the target “medium” are expected to result from manufacturing inaccuracies.

In Figure 17, the predicted and measured absorption coefficient data is shown for all three target curves. Similar to the corresponding plot for the KNN model, the blue curve shows the target curve, the orange curve shows the predicted absorption coefficient and the green curve shows the measured absorption coefficient of the manufactured material. The optimization error is given in the plot’s captions. For the target curve “low”, see Figure 17a, a good agreement of all shown curves can be seen. The optimization error with a value of 0.001 is very low, this means that the ANN model can compute suitable geometry parameters for this design. Furthermore, the measured data are in very good agreement with the predicted absorption coefficient curve as well, thus the predicted geometry parameters prove appropriate for the practical application. For this target curve as well as for the others, the “kink” in the course of the absorption coefficient curve, already seen for the KNN model, can be observed as well and is expected to be a result of resonator effects caused by air-filled voids between the specimen and the impedance tube’s walls.

A rather undesirable result is shown in Figure 17b for the target curve “medium”. For instance, the predicted absorption coefficient curve (orange) is in good agreement with the target curve (blue), this is confirmed by the low optimization error of 0.002. Nevertheless, the manufactured specimen does not follow the designed material, the absorption coefficient is rather low with a maximum of approx. 0.66 at 5200 Hz, whereas the predicted absorption coefficient should have been 0.89 at this frequency. A specific reason for this deviation yet remains unknown. Compared to the KNN model design, it can be stated that the value of the bar spacing predicted using the ANN model is rather high (ANN model: 0.66 mm; KNN model 0.44 mm). Nevertheless, it is expected that the relations between the geometry and the resulting Biot parameters/the absorption coefficient are highly nonlinear, hence simple value comparisons for single values might not be feasible here. This issue could be thought of as a negative drawback from using ML models. Since ML models rely on learned relations only rather than implementing physical models, the interpretability of the model outputs is rather limited.

For the target curve “high”, the results are shown in Figure 17c. Here again, a rather good agreement of all three curves is observed. The optimization error between the target curve and the predicted absorption coefficient with a value of 0.002 is equivalently low as for the other target curves. Furthermore, the measurement data follow the predicted curve rather well, nevertheless, some deviations can be observed. For example, the absorption maximum is shifted approx. 200 Hz to lower frequencies and the declining of the absorption coefficient for frequencies above the absorption maximum is more strongly present as it is with the design data. It might be the case as well that here a physical limit is reached for which the applied lattice-style absorber design is not able to generate such high absorption coefficients for high frequencies. Nevertheless, the observed deviations are accepted to be rather small and the procedure in total seems to prove successful.

Regardless of the deviations in the high-frequency range, Figure 17c shows a very interesting behavior that should be hereafter discussed. Recalling the definition of the target design curves in Figure 14, it can be observed that the target curves “medium” and “high” leave the learned data range for high frequencies. Hence, to generate absorber designs that show an absorption coefficient comparable to these target curves, the applied ML models need some ability to extrapolate beyond the learned data range. For the KNN model, it could be shown that this model type does not have this capability. The ANN model, on the other side, shows this capability to a reasonable extent—the designs (orange curves) for the target curve “medium” and “high” follow the target curves and thus leave the learned data range. Moreover, at least the design for the target curve “high” shows that the design parameters are rather reasonable as the measured data as well leaves the learned data range.

### 3.4. Design of a Material with Different Height and Prediction of Another Frequency Range

The ML models are built to predict the Biot parameters from the specimen’s micro-scale geometry. Thus, material parameters of the porous material are obtained. The “detour” via the Biot parameters opens the opportunity to design other specimens with varying macro-scale geometries, such as the specimen height. Moreover, the possibility to predict the acoustic behavior for other application cases, for example other frequency ranges becomes possible. This is due to the fact that Biot parameters are material-specific parameters and thus no mixing of the material description and the acoustic behavior of the material in specific conditions (sound field, mounting of the specimen) occurs. Therefore is assumed that, if the Biot parameters identified on specimens with a small height can be used as well to predict the behavior of specimen with a larger height, the Biot parameters can be trusted as they give a reasonable description of the material.

**Figure 18:**
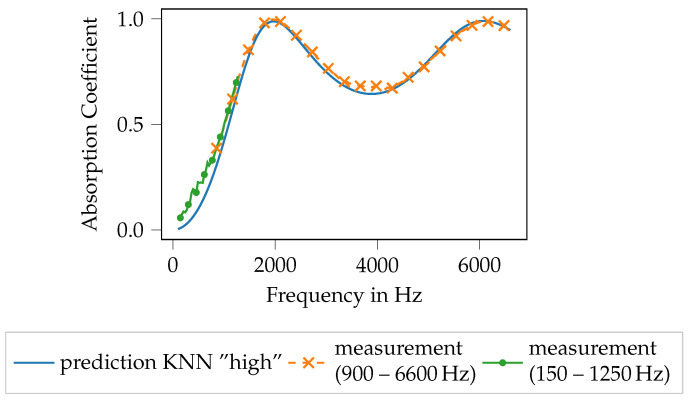
Computed and measured absorption coefficient of a specimen with a height l=30 mm and enlarged frequency range 150–6600 Hz. The computation employs the Biot parameters obtained from the KNN model for traget curve “high” and computes the absorption coefficient using the Equations (1)–(4), (A1) and (A2) for the new specimen height.

In order to verify this assumption, the design parameters of the specimen with target curve “high”, computed with the KNN model are used, see Table 7. Again, the ML model is used to compute the Biot parameters and the resulting absorption coefficient is computed using the Equations (1)–(4), (A1) and (A2) for a new specimen height l=30 mm. The specimen is manufactured using the design parameters and the resulting absorption coefficient is measured. Unlike for the initial specimen geometry, here the tested frequency range is enlarged to 150–6600 Hz. The results are shown in Figure 18.

In Figure 18 it can be seen that the computed and measured absorption coefficient coincide very well. This holds for both, the now strongly changed course of the absorption coefficient as well as the prediction in the low frequency regime. It is assumed that such a result could not have been obtained by means of ML alone. Therefore, the specimen height and the enlarged frequency range would have needed to be included in the input data, which here is not the case. Accordingly, many more samples would have been necessary to be produced in order to cover the larger dimension of the design space properly. This has been circumvented by computing the Biot parameters, which are material-specific parameters. Thus, the indeed larger effort using the Biot parameters on the other side can be viewed as a means of dimensionality reduction for the input data as well. Moreover, the obtained Biot parameters can be used in subsequent analyses: the Biot parameters are invariant to the type of sound field and mounting conditions of the material. Thus, this material-based description can be used for computations in other field types (diffuse field, varying angle of the incident waves, other frequency range as shown here, etc.) or even more complex analyses such as wave-resolving finite element computations as well. This could not be covered with the direct computation of the absorption coefficient from the ML models since those data are limited to the conditions with which the training data are obtained, here a planar normal incident sound field with a specific frequency range.

## 4. Conclusions and Outlook

The presented work aims to bridge the gap between the micro-scale geometry and acoustic material parameters of porous absorbers to allow a reasonable absorber design. The core challenge is that porous absorbers can be characterized from an acoustics point of view utilizing the Biot parameters and by geometric dimensions on the micro-scale. These two “worlds” are separated, the estimation of the Biot parameters from the geometry is possible only for simple special cases. The work presented here shows a way of connecting these two “worlds” through machine learning. In the following, the work is briefly summarized and an outlook on future works is given.

### 4.1. Conclusions

The presented work investigates a generic test specimen constructed by a micro-lattice of parallel bars. The specimens are manufactured using additive manufacturing, a specifically appropriate production procedure for porous materials. In order to connect the micro-scale geometry and the acoustic material parameters, namely the Biot parameters, the range of reasonable and manufacturable geometry values is sampled using a Latin Hypercube Strategy and an amount of 50 specimens is manufactured. The resulting specimens are optically and acoustically investigated, the latter comprising measurements of the absorption coefficient and flow resistivity. The Biot parameters of the specimens are derived using an inverse parameter identification scheme. Using the geometry of the specimens as inputs (features) and the Biot parameters as outputs (labels) two Machine-Learning regression models (K-Nearest Neighbors (KNN) and Artificial Neural Network (ANN)) are built and trained by means of supervised learning. The resulting models are then applied to a material design procedure with the goal of generating printable absorber structures that exhibit predefined absorption characteristics. This procedure finally is capable of computing the micro-scale geometry of the specimens that is required to obtain a porous absorber that shows the desired acoustic behavior, which is measured here in terms of the absorption coefficient.

The approach of using the Biot parameters here to design porous absorbers is chosen, as it exhibits some favorable implications. The Biot parameters can be viewed as material-specific parameters of the porous material and thus are invariant to the macro-geometry (for example the thickness) of the material, the mounting conditions or the sound field. If a quantity describing the behavior of the material was considered, for example, the absorption coefficient, a mixing of material description and material behavior would occur. This mixing is circumvented by predicting the Biot parameters directly and thus, these parameters can be used for other subsequent analyses as well without the limitation on how the parameters were obtained before.

A first aspect of the work presented here is the additive manufacturing of the specimen geometry, especially on the micro-scale. Here, a material extrusion process is employed, thereby specific means as targeted under-extrusion and the use of a 0.20 mm nozzle prove capable of reliably producing the required fine microstructures. This is verified using optical inspection using a profilometer. It can be shown that only small deviations of the desired and actually manufactured geometry exist. The acoustic inspection using the flow resistivity and absorption coefficient deliver the inputs for the inverse parameter identification procedure to obtain the Biot parameters. A first correlation analysis of the flow resistivity and the micro-scale geometry parameters of the specimen reveals that no trivial dependency between these parameters can be found. This finding motivates the use of machine-learning to exploit underlying dependencies that are coded in the data. Another important ingredient for the work presented here is the inverse parameter identification procedure that is used for obtaining the Biot parameters. Therefore, the specimens are modeled using a mechanical model for porous media, here the Johnson–Champoux–Allard–Lafarge (JCAL) model. This model takes the Biot parameters of the specimen as inputs. With this model, the absorption coefficient of the specimen can be computed for the case of mounting the specimen in front of an impervious rigid wall, which is the case for the measurements in the impedance tube that has been used here. Using the JCAL model, the inverse parameter identification procedure is set up using an evolutional algorithm. The results of the inverse identification of the Biot parameters are compared to analytical estimates and found to be trustworthy.

The machine-learning models are set up using the micro-scale geometry of the specimens as input and the Biot parameters as outputs. A correlation analysis shows that the models investigated here, namely the K-Nearest Neighbor model and the Artificial Neural Network are able to reasonably compute the Biot parameters from the specimen geometry. The obtained models are then applied to design porous absorbers that show a predefined acoustic behavior, here characterized by the absorption coefficient. Therefore, target curves of the absorption coefficient are defined and the specimen’ micro-scale geometry is inversely computed. It can be shown, that both models are able to generate printable absorber structures. However, some differences can be observed. A major difference between the models is their ability to extrapolate from the trained data—the KNN model is not able to produce specimens beyond the initial population, whereas the ANN model is able to compute designs that lie outside the initially learned data range. However, the measured absorption coefficient of the designed specimens matches the prediction in all three cases for the KNN model, the ANN model is able to predict the absorption coefficient only for two of three cases correctly. Finally, the procedure is applied to predict and generate another specimen with a different macro-geometry (here, the specimen height) as well. It can be shown that this application case can be handled by the proposed procedure fairly well.

Summarizing, the procedure to generate machine-learning based models that predict Biot parameters from porous absorbers micro-scale geometry can be viewed as successful. It was possible to generate models that are capable of the intended task and that these models can be successfully applied to design new absorbing structures. The higher effort for obtaining Biot parameters and thus focusing on the material description rather than on the acoustic behavior allow the application of the procedure for a multitude of practical applications, such as the generation of input parameters for finite element analyses or the idea of tailoring materials specifically for their intended application case.

### 4.2. Outlook

Although the presented process already delivers good results, some open questions remain. These will be hereafter discussed. For instance, the design of the presented work is rather complex, which is mainly due to the necessary computation of the Biot parameters. Therefore, it would be feasible to re-conduct the study and to measure more of the Biot parameters directly. This would result in a lower dimension of the optimization problem and probably more accurate results of the inverse parameter identification of the remaining Biot parameters. Another aspect for future works is the fact that not all prediction results of the machine-learning models could be verified by measurements. For example, it should be clarified why the result obtained with the ANN for the target curve “medium” shows high deviations compared to the other results. Moreover, it is assumed that the specimen design induces some physical limit that prevents from obtaining an even better acoustic behavior. The exploration of these limits is also subject to future work.

Another aspect regarding the application of the proposed method is the exploration of the physical relation of the Biot parameters and the specimen’s geometry. As already stated, for some special cases the Biot parameters can be estimated from the material micro-scale. This will be further investigated using the models obtained within this work and indeed is planned as the next step.

There is also a need for further research in the field of additive manufacturing processes, for example in the field of microstructures. An extended understanding of the dependencies between process parameters and the resulting microstructure is important. This knowledge would also establish the connection between deviations in the shape and form of the microstructure and resulting acoustic properties. The derivation of design rules for the manufacturing of microstructures utilizing material extrusion is necessary to ensure the realization of structures that are as small as possible. The topic of robust additive manufacturing of microstructures also has great research potential.

**Figure 19:**
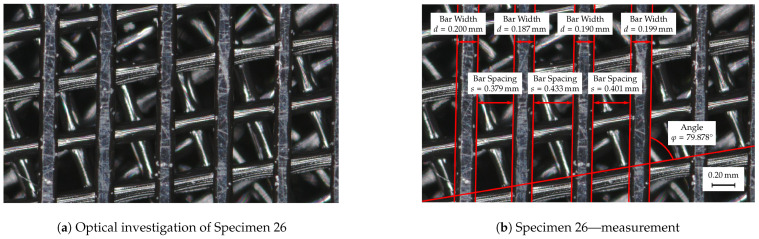
Actual-target comparison of specimen 26 with the Keyence VR5200 3D surface profilometer. (**a**) shows specimen 26 without measurement lines at 80× magnification. (**b**) shows specimen 26 with measurement lines and only slight deviations in the actual-target comparison

In addition, there is also a need for further research in the area of the resulting dimensional accuracy and also the shape of the additively manufactured microstructures. Figure 19 shows test specimens 26, whose bars have a bar width of 0.20 mm, which means that only one strand is needed to realize the bar width. In contrast to Figure 8, the test specimens 2 and 39 consists of bars with a bar width of 0.40 mm, which means that two parallel strands are required to realize the bar width. Figure 19 shows that the test specimens with only one strand for the realization of the bar width result in more homogeneous results in the geometric expression of the strands. Specifically, the one-strand design allows a much more constant application of the strands without fluctuating cross-sections. Furthermore, the actual-target comparison of specimen 26 shows desired dimensional results after the manufacturing process with only minor deviations. The reason for the poorer geometric expressions in the two-strand design may lie in the individual process parameters such as the cooling of the specimen during layer application or the nozzle temperature, but this needs to be investigated in further studies.

## Figures and Tables

**Table 1:** Overview of the design variables (see Figure 2a) and their variation ranges.

Bar Width (d)	Bar Spacing (s)	Bar Height (h)	Plane Angle (φ)
0.10–0.50 mm	0.10–1.00 mm	0.05–0.20 mm	0${}^{\circ }$–90${}^{\circ }$,

**Table 2:** Process parameters used in additive manufacturing.

	Nozzle Diameter
	0.20 mm
Nozzle temperature (${}^{\circ }$C)	210
Bed temperature (${}^{\circ }$C)	60
Layer height ${}^{1}$ (mm)	0.05–0.11
Flow (%)	85
Extrusion Speed (mm/s)	36
Cooling (%)	40
Outline direction	Inside-Out
Extrusion width (mm)	0.15; 0.20; 0.25

^1^ Increment of 0.005 mm.

**Table 3:** Scaling of the Biot parameters to balance out the different magnitudes.

Biot Parameter	Scaling	Biot Parameter	Scaling
$\Xi^{1}$	$\Xi^{★}=\Xi$	$\phi^{2}$	$\phi^{★}=1\times {\mathrm{10}}^{3}\;\phi$
${\alpha_{\infty }}^{3}$	$\alpha_{\infty }^{★}=1\times {\mathrm{10}}^{3}\alpha_{\infty }$	$\Lambda^{4}$	$\Lambda^{★}=1\times {\mathrm{10}}^{6}\;\Lambda$
${\Lambda^{\prime }}^{5}$	$\Lambda^{\prime^{★}}=1\times {\mathrm{10}}^{6}\;\Lambda^{\prime }$	${k_{0}^{\prime }}^{6}$	${k_{0}^{\prime }}^{★}=1\times {\mathrm{10}}^{12}\;k_{0}^{\prime }$

^1^ flow resistivity, ^2^ porosity, ^3^ tortuosity, ^4^ thermal characteristic length, ^5^ viscous characteristic length, ^6^ static thermal permeability.

**Table 4:** Training and test scores of the KNN and ANN model; uncertainty measure is two times the standard deviation of the cross-validation process (equiv. to approx. 95% confidence interval).

$R^{2}$-Score	KNN ${}^{1}$	ANN ${}^{2}$
cross-validation	0.80 ± 0.10	0.73 ± 0.13
test on unseen data	0.76	0.80

^1^ K-Nearest Neighbor, ^2^ Artificial Neural Network.

**Table 5:** Nominal geometry parameters of the specimens.

	Bar Width (d)	Bar Spacing (s)	Bar Height (h)	Angle (ϕ)
Specimen 2	0.40 mm	0.40 mm	0.08 mm	60.00 ${}^{\circ }$
Specimen 39	0.40 mm	0.80 mm	0.15 mm	70.00 ${}^{\circ }$

**Table 6:** Bounds for the evolutional optimization algorithm during absorber design.

Quantity	Lower Bound	Upper Bound
bar width (mm)	0.15	0.50
bar height (mm)	0.10	0.30
bar spacing (mm)	0.10	1.00
plane angle (${}^{\circ }$)	30	90

**Table 7:** Design parameters and measured values of the specimens designs using the KNN model. The printed specimens are measured using a 3D surface profilometer, quantities marked with a dash (’-’) could not be measured due to the measurement principle.

	Target “low”		Target “medium”		Target “high”	
Quantity	Design	Measured	Design	Measured	Design	Measured
bar width (mm)	0.17	0.21	0.26	0.24	0.20	0.19
bar height (mm)	0.12	–	0.11	–	0.15	–
bar spacing (mm)	0.93	0.89	0.44	0.41	0.33	0.325
plane angle (${}^{\circ }$)	63.40	63.399	63.000	63.256	33.40	33.466

**Table 8:** Design parameters and measured values of the specimens designs using the ANN model. The printed specimens are measured using 3D surface profilometer, quantities marked with a dash (‘-’) could not be measured due to the measurement principle.

	Target “low”		Target “medium”		Target “high”	
Quantity	Design	Measured	Design	Measured	Design	Measured
bar width (mm)	0.15	0.16	0.17	0.162	0.15	0.16
bar height (mm)	0.12	-	0.20	-	0.30	-
bar spacing (mm)	0.89	0.843	0.66	0.665	0.10	0.11
plane angle (${}^{\circ }$)	78.00	78.107	30.00	30.296	62.40	61.927

## Data Availability

Data sharing is not applicable to this article.

## Abbreviations

The following symbols and abbreviations are used in this manuscript:

*A*	specimen cross sectional area (flow resistivity measurement)
*d*	bar width
*h*	bar height
*k*	number of neighbors in KNN model
$k_{0}^{\prime }$	static thermal permeability
*l*	specimen height
*q*	volume flow through the specimen (flow resistivity measurement)
*R*	airflow resistance
*R*	coefficient of determination (performance measure of ML model training)
*s*	bar spacing
α	absorption coefficient
$\alpha_{\infty }$	tortuosity (high frequency limit)
Δp	pressure drop over the specimen (flow resistivity measurement)
Λ	viscous characteristic length
$\Lambda^{\prime }$	thermal characteristic length
Ξ	flow resistivity
ρ	Pearson’s Correlation Coefficient
φ	plane angle
ϕ	porosity
AM	additive manufacturing
ANN	artificial neural network
DOE	design of experiment
JCAL	Johnson–Champoux–Allard–Lafarge
KNN	k-nearest neighbor
LHS	latin hypercube sampling
MEX	material extrusion
ML	machine learning
PBF-P	powder bed fusion of polymers
PET-G	glycol modified polyethylene terephthalate
PLA	polylactide
VAT	vat photopolymerization

## Appendix A. Overview of the Design Parameter Space LHS Sampling

**Table A1:** Overview of the generated test Spec.s. The design parameters (*d*, *s*, *h*, φ) were varied using a Latin hypercube sampling. The process parameters (layer height, the extrusion width) are used during the manufacturing process and are given here for information only.

	Variation Parameters (Spec.)				Process Parameters (AM)	
	Bar Width (d)	Bar Spacing (s)	Bar Height (h)	Angle (φ)	Layer Height	Extrusion Width
Spec. 0	0.20 mm	0.70 mm	0.16 mm	40.00 ${}^{\circ }$	0.08 mm	0.20 mm
Spec. 1	0.50 mm	0.40 mm	0.11 mm	70.00 ${}^{\circ }$	0.11 mm	0.25 mm
Spec. 2	0.40 mm	0.40 mm	0.08 mm	60.00 ${}^{\circ }$	0.08 mm	0.20 mm
Spec. 3	0.40 mm	0.80 mm	0.06 mm	70.00 ${}^{\circ }$	0.06 mm	0.20 mm
Spec. 4	0.50 mm	0.20 mm	0.12 mm	80.00 ${}^{\circ }$	0.06 mm	0.25 mm
Spec. 5	0.30 mm	0.90 mm	0.15 mm	50.00 ${}^{\circ }$	0.075 mm	0.15 mm
Spec. 6	0.30 mm	0.70 mm	0.13 mm	50.00 ${}^{\circ }$	0.065 mm	0.15 mm
Spec. 7	0.20 mm	0.30 mm	0.17 mm	40.00 ${}^{\circ }$	0.085 mm	0.20 mm
Spec. 8	0.40 mm	0.50 mm	0.11 mm	80.00 ${}^{\circ }$	0.11 mm	0.20 mm
Spec. 9	0.30 mm	0.90 mm	0.10 mm	40.00 ${}^{\circ }$	0.10 mm	0.15 mm
Spec. 10	0.20 mm	0.90 mm	0.12 mm	50.00 ${}^{\circ }$	0.06 mm	0.20 mm
Spec. 11	0.30 mm	0.80 mm	0.07 mm	80.00 ${}^{\circ }$	0.07 mm	0.15 mm
Spec. 12	0.30 mm	0.50 mm	0.16 mm	80.00 ${}^{\circ }$	0.08 mm	0.15 mm
Spec. 13	0.50 mm	0.30 mm	0.14 mm	40.00 ${}^{\circ }$	0.07 mm	0.25 mm
Spec. 14	0.20 mm	0.90 mm	0.07 mm	30.00 ${}^{\circ }$	0.07 mm	0.20 mm
Spec. 15	0.30 mm	0.20 mm	0.06 mm	70.00 ${}^{\circ }$	0.06 mm	0.15 mm
Spec. 16	0.50 mm	0.80 mm	0.15 mm	80.00 ${}^{\circ }$	0.075 mm	0.25 mm
Spec. 17	0.20 mm	0.30 mm	0.08 mm	80.00 ${}^{\circ }$	0.08 mm	0.20 mm
Spec. 18	0.40 mm	0.20 mm	0.08 mm	60.00 ${}^{\circ }$	0.08 mm	0.20 mm
Spec. 19	0.20 mm	0.70 mm	0.07 mm	50.00 ${}^{\circ }$	0.07 mm	0.20 mm
Spec. 20	0.20 mm	0.70 mm	0.08 mm	70.00 ${}^{\circ }$	0.08 mm	0.20 mm
Spec. 21	0.20 mm	0.50 mm	0.11 mm	50.00 ${}^{\circ }$	0.11 mm	0.20 mm
Spec. 22	0.40 mm	0.70 mm	0.09 mm	50.00 ${}^{\circ }$	0.09 mm	0.20 mm
Spec. 23	0.30 mm	0.60 mm	0.18 mm	90.00 ${}^{\circ }$	0.09 mm	0.15 mm
Spec. 24	0.50 mm	0.60 mm	0.17 mm	70.00 ${}^{\circ }$	0.085 mm	0.25 mm
Spec. 25	0.50 mm	0.60 mm	0.06 mm	40.00 ${}^{\circ }$	0.06 mm	0.25 mm
Spec. 26	0.20 mm	0.40 mm	0.20 mm	80.00 ${}^{\circ }$	0.10 mm	0.20 mm
Spec. 27	0.20 mm	0.50 mm	0.16 mm	90.00 ${}^{\circ }$	0.08 mm	0.20 mm
Spec. 28	0.40 mm	0.50 mm	0.17 mm	40.00 ${}^{\circ }$	0.085 mm	0.20 mm
Spec. 29	0.30 mm	0.90 mm	0.13 mm	60.00 ${}^{\circ }$	0.065 mm	0.15 mm
Spec. 30	0.30 mm	0.30 mm	0.10 mm	40.00 ${}^{\circ }$	0.10 mm	0.15 mm
Spec. 31	0.40 mm	0.40 mm	0.09 mm	60.00 ${}^{\circ }$	0.09 mm	0.20 mm
Spec. 32	0.50 mm	0.60 mm	0.14 mm	60.00 ${}^{\circ }$	0.07 mm	0.25 mm
Spec. 33	0.30 mm	0.60 mm	0.14 mm	50.00 ${}^{\circ }$	0.07 mm	0.15 mm
Spec. 34	0.20 mm	1.00 mm	0.19 mm	80.00 ${}^{\circ }$	0.095 mm	0.20 mm
Spec. 35	0.40 mm	0.90 mm	0.10 mm	30.00 ${}^{\circ }$	0.10 mm	0.20 mm
Spec. 36	0.30 mm	0.70 mm	0.05 mm	70.00 ${}^{\circ }$	0.05 mm	0.15 mm
Spec. 37	0.40 mm	0.80 mm	0.13 mm	60.00 ${}^{\circ }$	0.065 mm	0.20 mm
Spec. 38	0.30 mm	0.90 mm	0.19 mm	90.00 ${}^{\circ }$	0.095 mm	0.15 mm
Spec. 39	0.40 mm	0.80 mm	0.15 mm	70.00 ${}^{\circ }$	0.075 mm	0.20 mm
Spec. 40	0.20 mm	1.00 mm	0.18 mm	90.00 ${}^{\circ }$	0.09 mm	0.20 mm
Spec. 41	0.30 mm	0.50 mm	0.09 mm	30.00 ${}^{\circ }$	0.09 mm	0.15 mm
Spec. 42	0.40 mm	0.40 mm	0.17 mm	50.00 ${}^{\circ }$	0.085 mm	0.20 mm
Spec. 43	0.40 mm	0.30 mm	0.20 mm	30.00 ${}^{\circ }$	0.10 mm	0.20 mm
Spec. 44	0.30 mm	0.60 mm	0.19 mm	70.00 ${}^{\circ }$	0.095 mm	0.15 mm
Spec. 45	0.20 mm	0.80 mm	0.18 mm	30.00 ${}^{\circ }$	0.09 mm	0.20 mm
Spec. 46	0.40 mm	0.40 mm	0.14 mm	80.00 ${}^{\circ }$	0.07 mm	0.20 mm
Spec. 47	0.20 mm	0.30 mm	0.06 mm	60.00 ${}^{\circ }$	0.06 mm	0.20 mm
Spec. 48	0.40 mm	0.40 mm	0.12 mm	40.00 ${}^{\circ }$	0.06 mm	0.20 mm
Spec. 49	0.30 mm	1.00 mm	0.12 mm	60.00 ${}^{\circ }$	0.06 mm	0.15 mm

## Appendix C. Inverse Parameter Identification for the Biot Parameters

**Table A2:** Settings of the evolutional algorithm used for the inverse parameter identification procedure. The implementation of the algorithm is used from the python library scipy [67] an follows [66].

Property/Parameter	Setting/Value
objective function	takes the Biot parameters, applies Equations (1)–(A2) to compute the absorption coefficient, computes the error using Equation (5) with regard to the measured results and returns the error
bounds	$\phi \in \left[0.20,0.80\right]$ $\alpha_{\infty }\in \left[0,10\right]$ $\Lambda \in \left[1\times {\mathrm{10}}^{-7},1\times {\mathrm{10}}^{-3}\right]$ $\Lambda^{\prime }\in \left[1e-7,1e-3\right]$ $k_{0}^{\prime }\in \left[1e-12,1e-8\right]$
convergence tolerance (rel.)	1 × 10^−2^
mutation parameter	0.50
recombination parameter	0.70
max. iterations	1000
population size	75

## Appendix D. Mathematical Formulation o the JCAL Model Used Here

The mathematical formulation of the JCAL model used within this work is shown in Equations (A1) and (A2), respectively. It is taken from [63]. Table A3 lists the used symbols and the described physical quantity.

(A1) $$\begin{array}{cc} \tilde{\rho } & =\frac{\alpha_{\infty }\rho_{0}}{\phi }\left[1+\frac{\Xi \phi }{j\omega \rho_{0}\alpha_{\infty }}\sqrt{1+j\frac{4\alpha_{\infty }^{2}\eta \rho_{0}\omega }{\Xi^{2}\Lambda^{2}\phi^{2}}}\right] \end{array}$$

(A2) $$\begin{array}{cc} \tilde{K} & =\frac{\frac{\gamma P_{0}}{\phi }}{\gamma -\left(\gamma -1\right){\left[1-j\frac{\phi \kappa }{k_{0}^{\prime }C_{p}\rho_{0}\omega }\sqrt{1+j\frac{4k_{0}^{\prime^{2}}C_{p}\rho_{0}\omega }{\kappa \Lambda^{\prime^{2}}\phi^{2}}}\right]}^{-1}} \end{array}$$

**Table A3:** Symbols used in the JCAL model.

Symbol	Quantity	Symbol	Quantity
Ξ	flow resistivity	η	dynamic viscosity of the fluid
$\alpha_{\infty }$	tortuosity	$\rho_{0}$	ambient density of the fluid
ϕ	porosity	ω	angular frequency
Λ	viscous characteristic length	$C_{p}$	heat capacity at constant pressure
$\Lambda^{\prime }$	thermal characteristic length	γ	heat capacity ratio
$k_{0}^{\prime }$	static thermal permeability	κ	thermal conductivity

## Appendix E. Settings of the Machine Learning Models

**Table A4:** Settings of the KNN and ANN model (implementation see python-library scikit-learn [71]).

**(a) Settings of the KNN model**	
**parameter**	**setting**
n neighbors	5
weights	distance (weighting by inverse of the distance)
algorithm	auto (default, chooses according input data)
leaf size	30 (default)
p	2 (default)
metric	minkowski (default)
metric params	None (default)
n jobs	Nonde (default)
parameter	setting
hidden layers	4000-100
activation	ReLU
solver	Adam
alpha	1 × 10^−4^ (default)
batch size	auto (default)
learning rate	constant (default)
learning rate init	1 × 10^−3^ (default)
power t	0.50 (default)
max iter	50.00
shuffle	True (default)
random state	None (default)
tol	1 × 10^−9^
verbose	False (default)
warm start	False (default)
momentum	0.90 (default)
nesterovs momentum	True (default)
early stopping	False (default)
validation fraction	0.10 (default)
beta 1	0.90 (default)
beta 2	1.00 (default)
epsilon	1 × 10^−8^ (default)
n iter no change	100
max fun	15,000 (default)

## Appendix G. Settings of the Evolutional Algorithm for the Inverse Absorber Design and Results

**Table A5:** Settings of the evolutional algorithm used for the inverse specimen design procedure. The implementation of the algorithm is used from the python library scipy [67] an follows [66].

Property/Parameter	Setting/Value
objective function	takes the design variables, inputs into ML model, computes Biot parameters, applies Equations (1)–(A2) to compute the absorption coefficient, computes and returns the error using Equation (8) with regard to target curve
bounds	see Table 6
convergence tolerance (rel.)	1 × 10^−2^
mutation parameter	0.50
recombination parameter	0.70
max. iterations	1000
population size	60
